# The interactions of monomeric acridines and unsymmetrical bisacridines (UAs) with DNA duplexes: an insight provided by NMR and MD studies

**DOI:** 10.1038/s41598-023-30587-y

**Published:** 2023-03-01

**Authors:** Tomasz Laskowski, Michał Kosno, Witold Andrałojć, Joanna E. Frackowiak, Julia Borzyszkowska-Bukowska, Paweł Szczeblewski, Nikola Radoń, Maria Świerżewska, Anna Woźny, Ewa Paluszkiewicz, Zofia Mazerska

**Affiliations:** 1 Department of Pharmaceutical Technology and Biochemistry and BioTechMed Centre, Faculty of Chemistry, Gdańsk University of Technology, Gabriela Narutowicza Str. 11/12, 80-233 Gdańsk, Poland; 2 Institute of Bioorganic Chemistry, Polish Academy of Sciences, Zygmunta Noskowskiego Str. 12/14, 61-704 Poznań, Poland; 3 Faculty of Applied Physics and Mathematics, Gdańsk University of Technology, Gabriela Narutowicza Str. 11/12, 80-233 Gdańsk, Poland

**Keywords:** Drug discovery and development, Solution-state NMR, Structure elucidation, Chemotherapy, DNA, Molecular dynamics

## Abstract

Members of a novel class of anticancer compounds, exhibiting high antitumor activity, i.e. the unsymmetrical bisacridines (UAs), consist of two heteroaromatic ring systems. One of the ring systems is an imidazoacridinone moiety, with the skeleton identical to the structural base of Symadex. The second one is a 1-nitroacridine moiety, hence it may be regarded as Nitracrine’s structural basis. These monoacridine units are connected by an aminoalkyl linker, which vary in structure. In theory, these unsymmetrical dimers should act as double-stranded DNA (dsDNA) bis-intercalators, since the monomeric units constituting the UAs were previously reported to exhibit an intercalating mode of binding into dsDNA. On the contrary, our earlier, preliminary studies have suggested that specific and/or structurally well-defined binding of UAs into DNA duplexes might not be the case. In this contribution, we have revisited and carefully examined the dsDNA-binding properties of monoacridines C-1305, C-1311 (Symadex), C-283 (Ledakrin/Nitracrine) and C-1748, as well as bisacridines C-2028, C-2041, C-2045 and C-2053 using advanced NMR techniques, aided by molecular modelling calculations and the analysis of UV–VIS spectra, decomposed by chemometric techniques. These studies allowed us to explain, why the properties of UAs are not a simple sum of the features exhibited by the acridine monomers.

## Introduction

A novel class of anticancer agents, i.e. unsymmetrical bisacridines (UAs), was recently synthesized and subjected to numerous studies^1^. They exhibited high antitumor activity against over a dozen tested cancer cell lines, as well as antitumor activity against Walker 256 rat adenocarcinoma and ten human tumor xenografts in nude mice. Notably, the compounds which displayed the highest activity strongly inhibited pancreatic cancer cell lines^1^. Studies on the biological effects of these compounds demonstrated their ability to suppress 3D cancer spheroid growth^2^. Additionally, their anticancer activity was enhanced when bound to quaternary quantum dots, resulting in selective upregulation of their cellular uptake^3^.

The molecular foundations of biological activity of UAs, as well as their potential molecular targets, ale still extensively researched. As far as we know, UAs are highly cytotoxic compounds with IC_50_ values in the ng/mL range, although sensitivity of individual cell lines to the compounds varies. Previous results established that cells treated with UAs undergo apoptosis or senescence^4^. It has been demonstrated that UAs rapidly enter the cell, as they are detected in the cells as early as 1 h after treatment^3^ (some results unpublished). However, the degree in which UAs are retained in the cell following their entry is markedly different for various cell lines. Time-dependent determination of UA concentration after prolonged incubation reveals either increased, decreased or constant concentration of UAs in cells^3^. Moreover, a pH-dependent localization of UAs in the cell has been observed, as UA concentration was increased in organelles characterized by low pH, such as lysosomes and endosomes^3,5^.

**Figure 1:**
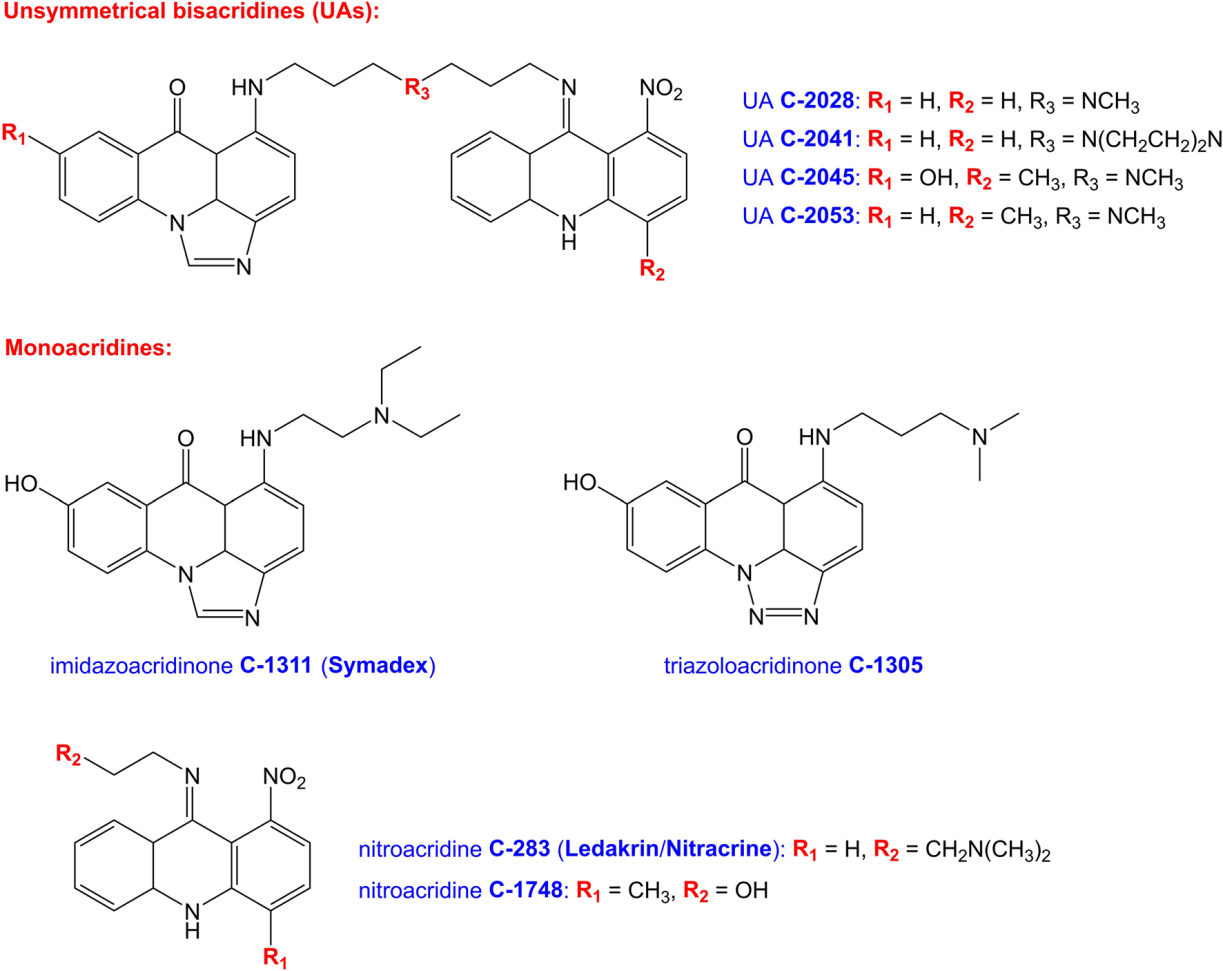
The structures of the examined compounds.

Members of the UA group share a common structural feature: they consist of two heteroaromatic ring systems, which are essentially acridine derivatives. One of the ring systems is an imidazoacridinone moiety, with the skeleton identical to the structural base of C-1311 (Symadex)^1,6^. The second one is a 1-nitroacridine moiety, optionally with methyl substituent at *para* position in relation to the–NO_2_ function. Hence, it may be regarded as having a C-283’s (Ledakrin/Nitracrine) or C-1748’s structural basis. These monoacridine units are connected by an aminoalkyl linker, which vary in structure (Fig. 1). Since the UA molecules contain numerous potential protonation sites, physicochemical properties and the resulting antitumor activity of these compounds might be highly dependent on the pH of the environment, which was unambiguously proven by our previous report^6^.

Earlier studies have strongly suggested that C-283, C-1311 (Symadex) and C-1748 are the double-stranded (ds) DNA intercalators^7–12^. Recently, we have reported that Symadex is an efficient dsDNA intercalator with a preference to AG/CT or GA/TC dinucleotide steps^11,12^. The following studies on its analog, triazoloacridinone C-1305, have shown that the latter compound exhibited a notable sequence specificity, with the TA/TA dinucleotide step being the preferred binding site within double-stranded DNA^13^. Basing on these results and other, recent findings^14–16^, we have also suggested that the TA/TA sequence might act as a preferred binding site for many DNA intercalators, at least for the acridine-based ones and those which do not exhibit specific stereochemical requirements while binding to dsDNA. The TA/TA binding cavity was not previously considered in the case of imidazoacridinone C-1311, whereas the binding of C-283 and C-1748 to nucleic acids was never examined by means of NMR spectroscopy—neither structurally, nor regarding a potential sequence-specificity of these drugs.

One might expect that structures consisting of two acridine-based ring systems would be excellent dsDNA-binding agents as well. On the contrary, our previous studies have suggested that—surprisingly—unsymmetrical bisacridines (UAs) do not interact with DNA duplexes^1^. Considering all the above, in this contribution we have presented detailed, NMR- and UV–VIS-based studies on C-1305, C-1311, C-283 and C-1748, as well as UAs, binding to various sequences of double-stranded DNA. In this respect, the aim of our studies was to dive deep into the properties of the aforementioned acridine monomers in order to discuss, why the features exhibited by unsymmetrical bisacridines are far from being a simple sum of properties displayed by the monomeric units.

## Results

### Monomeric acridine derivatives—1D NMR studies

The palindromic duplexes (further referred to as **D1** to **D9**), examined in the presence of the monomeric acridine derivatives, were presented in Table 1. Those sequences—combined—included all 10 possible dinucleotide steps occurring in double-stranded DNA and were identical to the ones designed for studies on C-1305^13^. The interpretation of the resulting ^1^H NMR spectra was based on the same assumptions as presented in our previous work^13^.

**Table 1:** Examined intercalation sequence-specificity of C-1311 (Symadex), C-283 (Ledakrin/Nitracrine) and C-1748. Results for C-1305 were taken from previous studies^13^.

Oligonucleotide codename	Sequence 5′–3′	Dinucleotide steps binding C-1305^13^	Dinucleotide steps binding C-1311	Dinucleotide steps binding C-283	Dinucleotide steps binding C-1748
D1	CCCGGG	CG/CG	CG/CG	–	–
D2	CGATATCG	TA/TA	TA/TA	–	–
D3	CCCTAGGG	TA/TA	TA/TA	–	–
D4	GGGTACCC	TA/TA	TA/TA	–	–
D5	CCCATGGG	TG/CA	–	–	–
D6	GGGATCCC	–	–	–	–
D7	GTACGTAC	TA/TA	TA/TA	–	–
D8	CTAGCTAG	TA/TA	TA/TA	–	–
D9	GAACGTTC	CG/CG	–	–	–

In order to: (1) suppress monoacridines’ tendency to self-associate in aqueous solutions; and to (2) ensure a highest possible molar fraction of a single spectral form of a given ligand within the probe while interacting with nucleic acids, all the following studies were performed in cacodylate buffer at pH = 5.0 with a low NaCl concentration (10 mM). In these conditions, the examined dsDNA palindromes (Table 1) maintained standard A-DNA (**D3**, **D6**, **D7**^17,18^) or B-DNA conformations (the rest), whereas in the case of the studied monoacridines their dominant spectral form in a solution was a positively charged monomer (excluding C-1748, which does not contain any charge in these conditions). Notably, **D6** required a higher concentration of NaCl (50 mM) in order to assume a double-helix conformation in a solution.

#### Imidazoacridinone C-1311 (Symadex)

**Figure 2:**
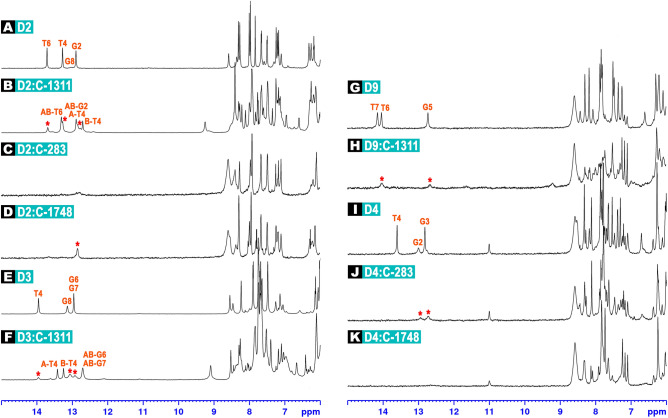
Exemplary ^1^H NMR spectra of: free **D2** duplex (**A**); **D2** duplex interacting with C-1311 (**B**); **D2** duplex interacting with C-283 (**C**); **D2** duplex interacting with C-1748 (**D**); free **D3** duplex (**E**); **D3** duplex interacting with C-1311 (**F**); free **D9** duplex (**G**); **D9** duplex interacting with C-1311 (**H**); free **D4** duplex (**I**); **D4** duplex interacting with C-283 (**J**); **D4** duplex interacting with C-1748 (**K**). For the explanation of duplex codenames, please consult Table 1. In all cases, the stoichiometry of the examined complexes was dsDNA/ligand 1:1.5 mol/mol. Red asterisks mark the imino protons of the remaining, free dsDNA duplexes.

Our ^1^H NMR assessments for C-1311, which were partially displayed at Fig. 2, have clearly evidenced that Symadex in fact prefers the TA/TA binding site over the previously postulated AG/TC and GA/CT dinucleotide steps^11,12^ (Fig. 2A–F). Considering the data gathered for all sequences examined herein, the sequence specificity of C-1311 was established as TA/TA >> CG/CG. Although a specific intercalation of Symadex into the TG/CA step could not be observed in a straightforward manner, the preferred 5′-pyrimidine-purine-3′ binding pattern was still visible in the case of C-1311, as it was for triazoloacridinone C-1305^13^. Also, we were unable to observe a specific binding of Symadex to AG/CT and GA/TC sites during our examinations. Whereas this fact could be associated with the alteration of the experimental conditions in comparison to our previous studies^11,12^, the main reason seemed to be the presence of the better options (TA/TA or CG/CG), which were not located at the ends of the palindromic sequences.

Two of the studied non-covalent adducts, d(CGATATCG)_2_:C-1311 (**D2L**) and d(CCCTAGGG)_2_:C-1311 (**D3L**), were selected for detailed 2D NMR studies for a direct comparison with the previously reported d(CGATATCG)_2_:C-1305 and d(CCCTAGGG)_2_:C-1305 complexes. Additionally, C-1311’s dissociation constants to palindromic NTAN tetranucleotide steps (where N stands for C/G/A/T nucleotides) were assessed using UV–VIS spectroscopy. The results of these studies were discussed in separate chapters.

#### Nitroacridines C-283 (Ledakrin/Nitracrine) and C-1748

The same **D1**–**D9** palindromic octamers were examined in the presence of C-283 and C-1748 (Table 1). Unfortunately, the resonances of T/G imino protons, effectively vanishing upon the addition of nitroacridine ligands (Fig. 2C,D,J,K), pointed to the unspecific DNA/ligand interactions. While in the case of C-1311 a new, arising set of DNA’s imino resonances was a testament of the formation of an intercalation complex (Fig. 2A,B,E,F), no such phenomenon was observed in the presence of neither C-283 nor C-1748 for any of the studied duplexes. Therefore, taking into account the evident broadening of the resonances of the aromatic H5/H6/H8 protons of the **D1**–**D9** duplexes (with no new DNA resonances arising, which would suggest the presence of a stereochemically defined DNA:ligand adduct, Fig. 2C,D,I–K) and considering the fact that—at this stage—the TA/TA dinucleotide step should be regarded as a default binding site of acridine-based intercalators, one should conclude that the studied nitroacridines—albeit unspecifically interacting with DNA—are not the most efficient dsDNA intercalators, which contradicts the current paradigm^7–10,19,20^.

In order to gain a deeper insight into the dsDNA:nitroacridine interactions, we have additionally performed titration experiments, using the d(CGATATCG)_2_ (**D2**) palindrome as a DNA host. Upon the addition of C-283 (Nitracrine, see Fig. S10A in the Supplementary Data), the urgent vanishing of T4 and T6 imino resonances was observed, whereas the G2 and G8 imino proton resonances remained basically unchanged, until the DNA:C-283 1:1 mol/mol stoichiometry of the solution was reached. Also, A3H2 and A5H2 aromatic protons were gradually widening, as well as T4H6 and T6H6; and T4CH_3_ and T6CH_3_ resonances. Notably, the rest of the resonances were only slightly shifted or—in most cases—remained unaltered. Further addition of the ligand—in order to exceed the 1:1 stoichiometry—resulted in an image similar to Fig. 2C.

Since all the aforementioned protons are located at the central region of the **D2** helix, the titration experiments have proven that C-283 interacted with **D2** at the centre of the studied octamer, close to a potential TA/TA binding cavity. However, as we did not observe an appearance of a new set of DNA resonances—only the broadening of some among them (while the ligand resonances themselves were broadened beyond detection), the DNA:ligand complex formation (and dissociation) must occur on the fast-to-intermediate exchange regime on the chemical shift timescale. As all our previous experience with intercalative binding of acridine derivatives to DNA^13^ suggests complex lifetimes within the slow exchange regime, we interpret such a result as an effect of a more shallow and perhaps less spatially defined mode of interaction, presumably with the minor groove of DNA. It must be noted that the intercalation mode of action of Nitracrine cannot be excluded; moreover—the apparent broadening of thymine methyl resonances suggests, that the intercalation event might possibly occur. Nevertheless, even if it occurs, the stability of the resulting dsDNA:C-283 intercalation complexes is orders of magnitude lower in comparison to the complexes formed by imidazoacridinones and triazoloacridinones, i.e. C-1311 and C-1305.

In the case of **D2**:C-1748 titration experiments, in the end, the T4 and T6 imino resonances were notably weakened (yet still observable and sharp), whereas the A3H2, A5H2, T4H6, T6H6, T4CH_3_ and T6CH_3_ resonances were slightly broadened (Fig. S10B). The rest of the resonances remained unaltered. These results have suggested a DNA:ligand mode of interactions similar to the one proposed for C-283, albeit the nitroacridine C-1748 displayed far less pronounced affinity to **D2** palindrome.

### 2D NMR structural studies on d(CGATATCG)_2_:C-1311 (D2L) and d(CCCTAGGG)_2_:C-1311 (D3L) complexes

^1^H NMR assessments have indicated that imidazoacridinone C-1311 (Symadex) formed very well-defined 1:1 mol/mol non-covalent adducts with palindromes d(CGATATCG)_2_ (**D2**) and d(CCCTAGGG)_2_ (**D3**) (Fig. 2A,B,E,F, Table 1). Since the same octamers were chosen for structural studies on complexes formed by triazoloacridinone C-1305^13^, we’ve decided to directly compare the stereochemical features of the resulting adducts to the ones reported before.

The complexes formed by Symadex were examined in the same experimental conditions as the adducts formed by C-1305, hence the assignments of the resonances of the ligand-free **D2** and **D3** palindromes were taken from our previous study^13^. The assignments to the protons of the **D2L** and **D3L** systems were listed in the Supplementary Data (Table S1, Fig. S4).

**Figure 3:**
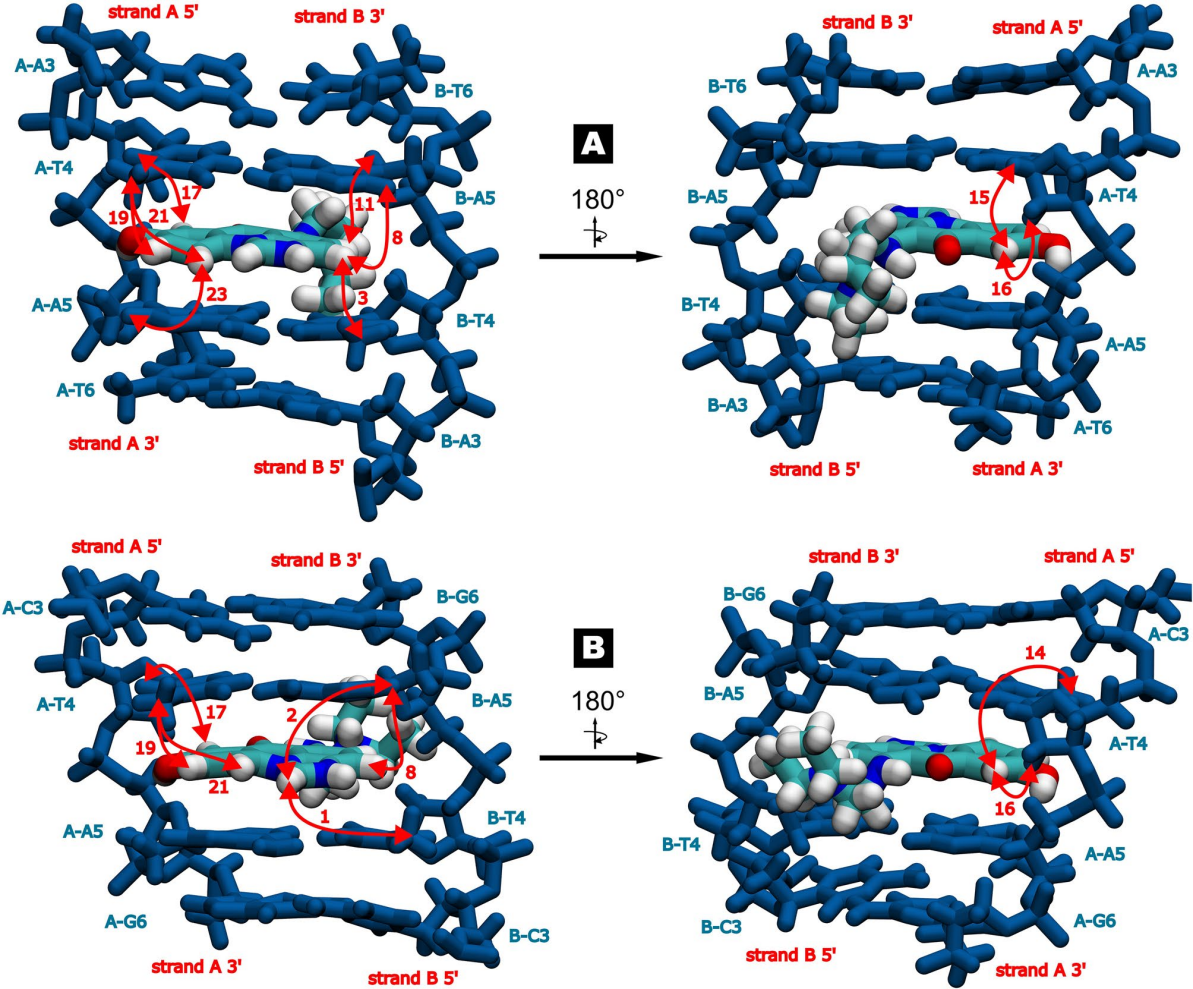
(**A**) The structure of the 5′-ATAT-3′ binding cavity with embedded ligand molecule, i.e. the central fragment of the d(CGATATCG)_2_:C-1311 (**D2L**) complex. (**B**) The structure of the 5′-CTAG-3′ binding cavity with embedded ligand molecule, i.e. the central fragment of the d(CCCTAGGG)_2_:C-1311 (**D3L**) complex. Selected DNA/ligand NOEs were depicted as red, bidirectional arrows, along with respective numbers corresponding to the correlations listed in Table 2.

Thorough examination of the NOESY spectra (Figs. S1 and S2) enabled an unambiguous location of the ligand between T4 and A5 moieties in the case of the both studied complexes. This was possible due to extensive sets of the observed DNA/C-1311 NOEs, listed in Table 2. These NOEs were later translated into input parameters for molecular dynamics calculations (MD), where they served as distance restraints. The resulting MD trajectories were finally subjected to cluster analysis in order to search for the most representative structures of each DNA/ligand adduct, which were displayed in Fig. 3.

**Table 2:** Observed d(CGATATCG)_2_:C-1311 (**D2L**) and d(CCCTAGGG)_2_:C-1311 (**D3L**) intermolecular NOE contacts. The intensities were classified as weak/medium/strong on the basis of the integration of the respective crosspeaks in the 2D NOESY spectra (τ_m_ = 150 ms) of the complexes and due to the lack of an internal standard of the DNA/ligand relaxation.

No.	C-1311 proton	DNA proton	D2L (intensity)	D3L (intensity)
NOE contacts involving aromatic protons of the ligand				
1	L-H1	B-T4CH_3_	–	+ (medium)
2	L-H1	A-A5H8	–	+ (medium)
3	L-H3	B-T4CH_3_	+ (weak)	–
4	L-H3	B-T4H1′	+ (medium)	+ (weak)
5	L-H3	B-T4H2′	+ (weak)	–
6	L-H3	B-T4H2″	+ (strong)	+ (medium)
7	L-H3	B-T4H6	+ (medium)	+ (weak)
8	L-H3	B-A5H8	+ (strong)	+ (medium)
9	L-H4	B-T4H2″	+ (strong)	+ (medium)
10	L-H4	B-T4H6	+ (medium)	+ (medium)
11	L-H4	B-A5H1′	+ (medium)	–
12	L-H4	B-A5H2′	–	+ (strong)
13	L-H4	B-A5H8	+ (medium)	+ (medium)
14	L-H7	A-T4CH_3_	+ (medium)	+ (medium)
15	L-H7	A-T4H1′	+ (medium)	+ (weak)
16	L-H7	A-T4H2″	+ (medium)	+ (medium)
17	L-H7	A-T4H6	+ (medium)	+ (medium)
18	L-H7	A-A5H8	+ (medium)	+ (weak)
19	L-H9	A-T4CH_3_	+ (strong)	+ (medium)
20	L-H9	A-A5H8	+ (strong)	+ (medium)
21	L-H10	A-T4CH_3_	+ (medium)	+ (medium)
22	L-H10	A-T4H1′	–	+ (medium)
23	L-H10	A-A5H8	+ (strong)	–

As it was reported before^11,12^, Symadex intercalated into the minor groove of the dsDNA palindromes. The hydroxyl group of the ligand, located at position 8, was prone to form a hydrogen bond with O4′ oxygen atom belonging to A5 deoxyribose moiety of the one of the dsDNA strands, which increased the stability of the resulting complex. This hydrogen bond existed for 82.2% of the simulation time in the case of **D2L** adduct and for 85.3% in the case of **D3L** ensemble. Notably, during the simulation time, the aminoalkyl sidechain of the ligand, albeit positively charged, did not exhibit any particular, preferred conformation within the minor groove of the DNA in the case of the both considered systems. This observation, while resulting from the MD calculations, actually had a solid, spectroscopic foundation: no NOEs between C-1311’s sidechain and the protons of the DNA were recorded in the NOESY spectra of either complex (Table 2). This was a major difference in comparison to the previous studies on C-1305, where its sidechain aligned itself along the sugar-phosphate backbone of the DNA, occasionally switching its orientation between parallel and antiparallel to a given DNA strand. Those conclusions were strongly supported by the observed sidechain/DNA NOEs^13^.

### UV–VIS-based determination of C-1311’s (Symadex) and C-1305’s dissociation constants while interacting with various, palindromic NTAN tetranucleotide steps

Our earlier studies on C-1305 have proven that the sequence-specificity of this drug’s binding to dsDNA via intercalation should be considered in tetranucleotide steps^13^. NMR assessments have suggested that the CTAG binding site was preferred over the GTAC and ATAT sequences, whereas the exact values of C-1305’s dissociation constants from various NTAN sites were not established^13^. The tetranucleotide dsDNA specificity was also confirmed in the case of C-1311, yet the recorded NMR spectra have strongly suggested its notably different preferences in that regard (ATAT > CTAG, GTAC). In order to quantify these preferences, i.e. in a form of dissociation constants, we have designed four additional palindromic DNA octamers, **B1**–**B4**, containing all possible NTAN tetranucleotide steps with identical flanking CG/CG base pairs (Table 3).

**Table 3:** Microscopic dissociation constants of all possible palindromic NTAN tetranucleotide steps (N stands for C/G/A/T) binding C-1311 and C-1305, determined upon chemometric analysis of the UV–VIS spectra.

Oligomer codename	Sequence	Preferred binding site of the ligands	C-1311		C-1305	
			Relative affinity	Dissociation constant (μM)	Relative affinity	Dissociation constant (μM)
**B1**	CG**GTAC**CG	TA/TA	Medium	0.524	Strong	0.145
**B2**	CG**CTAG**CG	TA/TA	Strong	0.260	Strong	0.227
**B3**	CG**ATAT**CG	TA/TA	Strong	0.156	Medium	0.608
**B4**	CG**TTAA**CG	TA/TA	Very strong	0.034*	Strong	0.248

*This value was established upon only two experimental points and should be regarded to as an estimation.

DNA-binding affinity was estimated by means of UV–VIS spectroscopy. Thorough chemometric analysis of the resulting spectra has indicated that C-1311 did interact with all of the studied sequences, yet the binding affinities were considerably different within the studied set of dsDNA oligomers. The weakest binding of the ligand, i.e. the highest dissociation constant of the complex has been observed in case of the octamer d(CG**GTAC**CG)_2_ (**B1**), whereas its interaction with d(CG**CTAG**CG)_2_ (**B2**) and (CG**ATAT**CG)_2_ (**B3**) sequences was relatively strong—although the dissociation constant to **B3** was notably lower than the one calculated for **B2**. Interestingly, the strongest DNA/ligand interactions have occurred in the case of d(CG**TTAA**CG)_2_ (**B4**) palindrome, yet the value of the **B4**:C-1311 dissociation constant was merely assessed, as it was impossible to quantify in a straightforward manner. In order to paint a bigger picture, we have also finally established the binding affinities of C-1305 in the presence of the same four **B1**–**B4** palindromes (Table 3), which—as expected—turned out to be notably different in comparison to the ones calculated for Symadex. More details on the conducted UV–VIS studies and the chemometric decomposition of the resulting spectra were given in the Supplementary Data (Figs. S5–S9).

Additionally, we have also performed UV–VIS studies on nitroacridines (C-283 and C-1748) interacting with short dsDNA palindromes. The results (data not shown) have strongly supported the claim that Nitracrine and C-1748 bind to DNA in a rather unspecific manner. The dissociation constants were impossible to determine, since the ligands’ spectra basically remained unchanged upon the titration of the DNA palindromes.

### A proposal of the optimal palindromic dsDNA octamer acting as an intercalator-trap

Taking into account the results obtained for both C-1305 and C-1311 regarding their affinities to the palindromic NTAN tetranucleotide steps, the 5′-pyrimidine-T-A-purine-3′ (5′-Pyr-T-A-Pu-3′) sequences generally seem to be in favor while compared to the 5′-Pu-T-A-Pyr-3′ options. Among the former, the 5′-CTAG-3′ tetranucleotide step was dubbed to be a compromise between its affinity to ligand molecules and the easiness of the interpretation of its NMR spectra. Although the palindromic octamer d(CC**CTAG**GG)_2_ (**D3**) examined herein and during the previous studies^13^ contains CTAG sequence, it also incorporates CCC/GGG triads, which turned out to be very complicated to assign, due to severe superposition of proton resonances. In the end, whereas the **D3** sequence served as a good host for ligand binding, its spectroscopic description was quite challenging, especially in a ligand-bound state. Therefore, we have examined the d(CG**CTAG**CG)_2_ octamer (**B2**, see Table 3) as a potential ‘golden mean’, considering its affinity to acridine-base ligands and its accessibility in terms of 2D NMR assignments.

**Figure 4:**
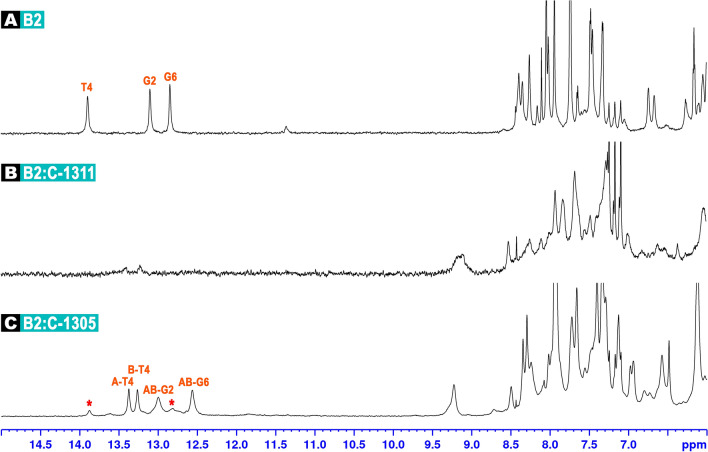
^1^H NMR spectra of: free palindromic duplex d(CGCTAGCG)_2_, codenamed **B2** (**A**); **B2** duplex interacting with C-1311 (**B**) and **B2** duplex interacting with C-1305 (**C**). In all cases, the stoichiometry of the examined complexes was dsDNA/ligand 1:1.5 mol/mol. Red asterisks mark the imino protons of the remaining, free dsDNA duplexes.

**Figure 5:**
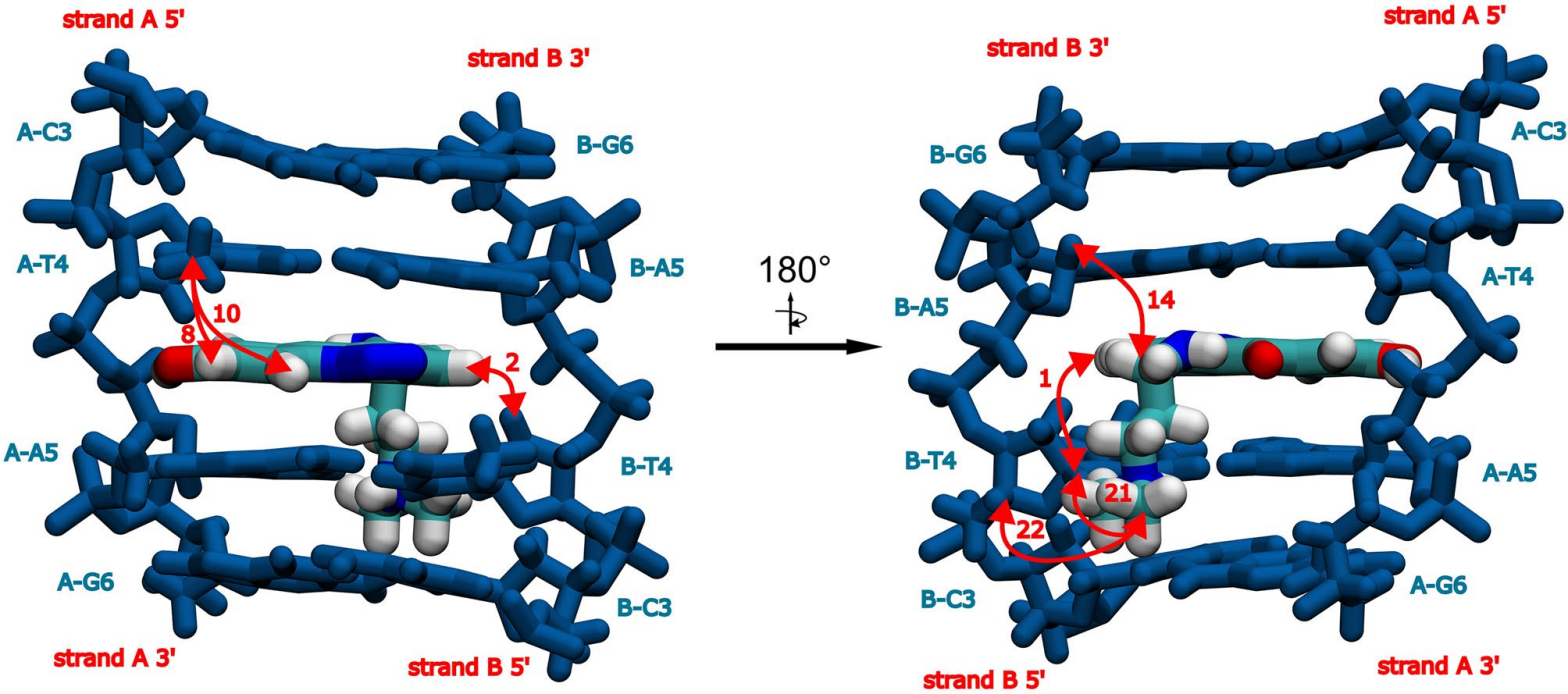
The structure of the 5′-CTAG-3′ binding cavity with embedded ligand molecule, i.e. the central fragment of the d(CGCTAGCG)_2_:C-1305 (**B2L**) complex. Selected DNA/ligand NOEs were depicted as red, bidirectional arrows, along with respective numbers corresponding to the correlations listed in Table 4.

In the case of C-1305, the resulting **B2**:ligand complex was very well defined, while the in-solution balance between free and bound state of the DNA was significantly shifted towards the complex formation (Fig. 4). 2D NMR studies conducted on d(CGCTAGCG)_2_:C-1305 (**B2L**) adduct have confirmed that a single ligand molecule has intercalated at the very centre of the **B2** octamer, yielding a non-covalent complex of a conformational properties very similar to the ones reported before^13^. This was a welcomed and expected result; more details on this structure were given at Fig. 5 and Table 4, as well as in the Supplementary Data (Tables S1 and S2, Fig. S4). Notably, the assignments to the protons of the ligand-bound **B2** octamer (Fig. S3) were considerably more straightforward in comparison to the **D3** duplex (Fig. S2).

**Table 4:** Observed d(CGCTAGCG)_2_:C-1305 (**B2L**) intermolecular NOE contacts. The intensities were classified as weak/medium/strong on the basis of the integration of the respective crosspeaks in the 2D NOESY spectra (τ_m_ = 150 ms) of the complexes and due to the lack of an internal standard of the DNA/ligand relaxation.

No.	C-1305 proton	DNA proton	B2L (intensity)
NOE contacts involving aromatic protons of the ligand			
1	L-H3	B-T4H1′	Weak
2	L-H3	B-T4H2′	Medium
3	L-H3	B-T4H6	Medium
4	L-H7	A-T4CH3	Weak
5	L-H7	A-T4H1′	Medium
6	L-H7	A-T4H2″	Medium
7	L-H7	A-T4H6	Medium
8	L-H9	A-T4CH3	Medium
9	L-H9	A-T4H6	Strong
10	L-H10	A-T4CH3	Weak
11	L-H10	A-T4H1′	Strong
12	L-H10	A-T4H3′	Medium
NOE contacts involving protons of the aminoalkyl sidechain			
13	L-H15	A-T4H1′	Strong
14	L-H15	B-A5H1′	Medium
15	L-H16	A-T4H1′	Medium
16	L-H16	A-T4H6	Weak
17	L-H16	B-T4H1′	Weak
18	L-H16	B-T4H6	Weak
19	L-H17	A-T4H1′	Medium
20	L-H18	A-T4H1′	Medium
21	L-H18	B-T4H1′	Medium
22	L-H18	B-T4H3′	Weak
23	L-H18	B-A5H1′	Weak

On the contrary, while the UV–VIS studies on **B2**:C-1311 adduct have clearly indicated that a single **B2** duplex, as expected, hosted just one Symadex molecule (Fig. S9 and Table 3), the NMR examination of this system resulted in the spectra suggesting that the complex and the free DNA were in fact in an equilibrium exhibiting medium exchange regime (Fig. 4). This means that the timescale of the formation and the dissociation of the d(CGCTAGCG)_2_:C-1311 adduct was chemical shift differences between the bound and free states, yielding an averaged image consisting of severely broadened resonances. In the end, the **B2**:C-1311 system was not subjected to extensive 2D NMR experiments, since the alteration of the total and the relative concentrations of both **B2** palindrome and Symadex did not improve the resulting ^1^H NMR image.

### 1D NMR examinations of unsymmetrical bisacridines (UAs) interacting with dsDNA duplexes

**Figure 6:**
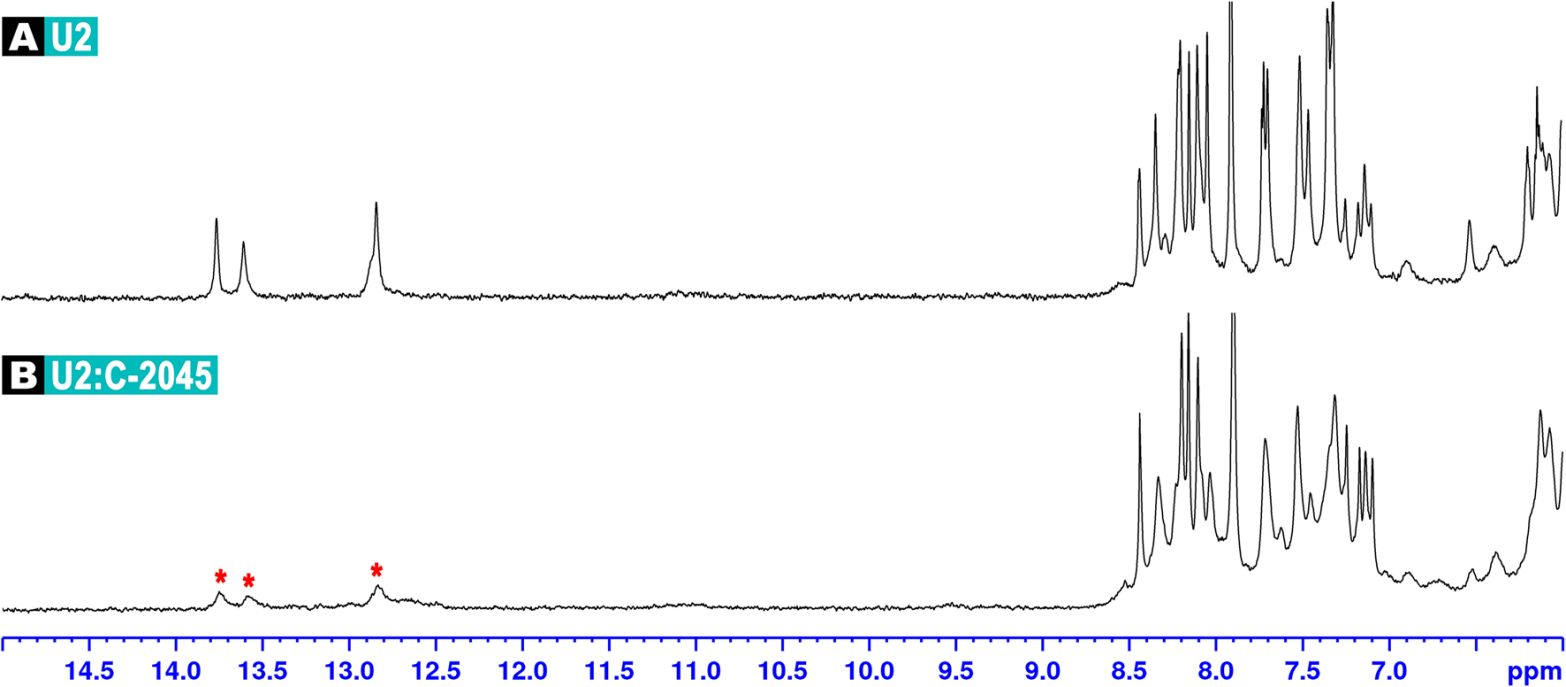
^1^H NMR spectra of: free palindromic duplex d(CGTAGCTACG)_2_, codenamed **U2** (**A**); **U2** duplex interacting with C-2045 (**B**). In all cases, the stoichiometry of the examined complexes was dsDNA/ligand 1:1.5 mol/mol. Red asterisks mark the imino protons of the remaining, free dsDNA duplexes. The **U2** was not analyzed by means of 2D NMR spectroscopy, hence the imino protons were not labelled.

Four unsymmetrical bisacridines (UAs): C-2028, C-2041, C-2045 and C-2053 were tested in the presence of specifically designed, longer palindromic sequences, containing TA/TA or TG/CA dinucleotide steps closer to the ends of the DNA duplexes (**U1**–**U4**, Table S3). During these experiments, the UAs did not intercalate into the aforementioned dsDNA oligomers at all. The resulting ^1^H NMR spectra of dsDNA:UA complexes were very similar to the ones produced by nitroacridine monomers (Fig. 6, please also consult Fig. 2), thereby suggesting unspecific DNA/ligand interactions, presumably in a minor groove of a helix. This observation was a confirmation of our previous assessments, revealing that UAs did not have a considerable impact on dsDNA melting points^1^. The source of this, at first glance, unexpected behavior of unsymmetrical bisacridines requires an in-depth discussion.

## Discussion

In this study, we have confirmed that imidazoacridinone C-1311 (Symadex) is an effective acridine-based dsDNA intercalator, which chooses the 5′-NTAN-3′ tetranucleotide sequence as a binding site, whenever available. The intercalation process occurs from the minor groove of the dsDNA helix, which is a result of the presence of positively charged aminoalkyl sidechain, which presumably serves as a molecular anchor, exhibiting affinity to the polyanionic sugar-phosphate backbone of the DNA strands. The 8-hydroxyl group of C-1311 (Fig. 1) is an additional structural element stabilizing the resulting complex, as it can form a hydrogen bond with the O4′ atom of the one the deoxyribose moieties. The formed non-covalent DNA:C-1311 adducts are relatively stable, although—as our experiments have revealed—they display different stability and spectroscopic properties in comparison to the complexes formed by C-1311’s triazoloacridinone cousin, i.e. C-1305. One of the main reasons for a notable disparity in binding affinities to dsDNA between these two, very similar compounds, must come from a major structural difference they exhibit, i.e. the structure of an aminoalkyl sidechain.

The NOESY spectra of the several DNA:C-1305 complexes studied to this end have always displayed NOE contacts between the protons of the ligand’s sidechain and the protons of the DNA. This fact has clearly indicated that C-1305’s aminoalkyl moiety exhibited some conformational preferences, i.e. it was prone to align itself along the sugar-phosphate backbone of a DNA strand. On the contrary, no similar NOEs were observed during the spectroscopic studies on the DNA:C-1311 adducts. Hence, it was concluded that the aminoalkyl sidechain of Symadex did not display any favoured alignments within a minor groove of the DNA duplexes. This outcome was additionally strengthened by the molecular modelling calculations of the examined systems. Whereas in the case of C-1305 the aminoalkyl moiety was clearly oriented—for the most of the simulation time—in a way that the spectra suggested, the sidechain of C-1311 did not exhibit any conformational agenda. The source of these differences lies within the structure of both moieties. C-1305’s sidechain consists of n-propyl and two methyl groups attached to a tertiary nitrogen, which is positively charged under the experimental conditions, whereas the C-1311’s moiety consists of three ethyl groups bound to a similar nitrogen atom. While the former is much more flexible, the latter is far more sterically hindered. Moreover, considering the position of the nitrogen atoms, the positive charge located at the sidechain of Symadex has a lower range, which prevents it from effective electrostatic interactions with the polyanionic DNA backbone after the intercalation occurs. Meanwhile, the positive charge of the aminoalkyl moiety of C-1305 has both better range and more conformational freedom, which enables it to act as a second anchor while bound to the DNA. The first anchor is the aforementioned ligands’ 8-OH–O4′ hydrogen bond inside the intercalation site. Although both C-1305 and C-1311 are able to exploit the hydrogen bond anchor after the intercalation event, the latter is effectively deprived of the second anchor, established by the interactions of the sidechain. This sidechain anchor introduces an additional stability to a resulting dsDNA:C-1305 non-covalent adduct. In the case of Symadex, the positively charged sidechain presumably plays a major, anchor role only at the possible (yet not evidenced experimentally) pre-intercalation stage, helping during the ligand’s settling within a minor groove of the dsDNA. In the end, while both C-1305 and C-1311 are very effective intercalators, the former one is able to create less dynamic complexes than the latter, due to the presence of an additional, stabilising factor.

Considering the data presented in Table 3, it is certain that the interactions between dsDNA backbone and ligand’s sidechain contribute to the observed dissociation constants, yet it is also quite clear that the second factor, contributing to the strength of DNA:ligand interactions, is the structure of the ligand’s ring system, since C-1305 and C-1311 exhibit a bit different electronic structures of their aromatic moieties (Fig. 1). One might conclude that, in general, C-1305 seems to create structurally less dynamic complexes, while C-1311 is a slightly stronger binder. Nevertheless, the dissociation constants of the both ligands also strongly depend on the bound dsDNA sequence, sometimes yielding inversed results. For instance, C-1305 binds significantly stronger to **B1** sequence in comparison to C-1311, whereas in the case of **B3** sequence the relation between C-1305 and C-1311 is ideally opposite, while compared to the dissociation constants established for **B1** (Table 3). Hence, although we may relate the structural dynamics of a complex to the sidechain’s structure, we actually cannot associate the DNA/ligand dissociation constants solely with the structure of a ligand’s sidechain, as it is an interplay of three factors: the sidechain’s structure, the structure of the ligand’s ring system and the structure of the binding cavity, i.e. the DNA host sequence.

Albeit numerous references have suggested that Nitracrine (C-283) and—by extent—its analogue, C-1748, are efficient dsDNA intercalators^7–10,19–22^, our examinations have not proven those claims. Nitroacridines are structurally based on acridine, a well-established dsDNA intercalating agent^23,24^, thus the assumption on their intercalation mode of action seemed very reasonable. However, the experimental support of this claim was based on the studies on longer fragments of digested cellular DNA, interacting with nitroacridines. The obtained data quite indirectly pointed to the possibility of intercalation, i.e. the melting point of the dsDNA was slightly raised in the presence of nitroacridines^21^, while the Nitracrine and its analogues induced unwinding of the supercoiled DNA and reversal of supercoiling, which is a characteristic feature of intercalating agents^22^. Notably, 3-nitroacridines exhibited stronger interactions with dsDNA in comparison to the 1-nitroacridines, i.e. C-283 (Nitracrine)^21^. Much more pronounced dsDNA:Nitracrine binding affinity could be observed after the metabolic activation of the drug, which was associated with the reduction of the 1-nitro moiety^25,26^. Presumably, this resulted in the formation of DNA/ligand covalent bonds and the DNA cross-linking^9,10^.

**Figure 7:**
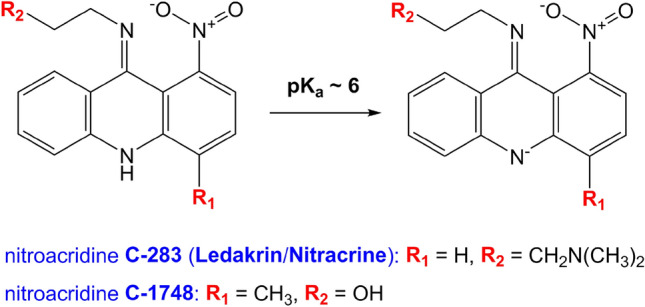
The deprotonation of the nitrogen atom, embedded in the acridine ring system. At pH below 7, the nitrogen inside the R_2_ moiety of C-283 is protonated and positively charged.

In the end, during our examinations, we could not prove a single intercalation event for neither C-283, nor C-1748, while studying several palindromic octamers. Even the TA/TA step, which is—considering the stacking energies—the easiest one to ‘get into’ from all 10 possible dinucleotide steps^27,28^, apparently was not inviting enough to create a stable intercalation complex with any of the discussed nitroacridines. Albeit we have observed some dsDNA/nitroacridine interactions, the resulting images were vastly different in comparison to the ones produced by C-1305 and C-1311. For these monoacridines, the spectra of their complexes displayed resonances of both free DNA and complex species, pointing to the slow exchange of the resulting adducts with free DNA and enabling the observation of DNA/ligand NOE contacts. In the case of C-283 and C-1748, the spectra revealed only one set of DNA resonances, whereas the ligand resonances could not be observed at all. Although the DNA:ligand spectra did, in fact, display the nitroacridines’ preference to locate themselves at the centre of a studied **D2** dsDNA duplex, yet the recorded proton resonances could not prove an intercalation mode of binding of neither Nitracrine, nor its C-1748 analogue. Our interpretation of the recorded spectra gravitates towards the non-intercalating minor-groove mode of DNA binding of both molecules, yet it must be stated that we cannot exclude the possibility of intercalation, displaying orders of magnitude higher dsDNA:ligand dissociation constant in comparison to C-1305 or C-1311. Notably, the C-283 (Nitracrine) affinity to a given dsDNA sequence was considerably higher than the one presented by C-1748. One could perhaps associate this observation with—again—the structure of the sidechains of the both nitroacridines. In one sentence, the sidechain of Nitracrine is longer and was positively charged in the given experimental conditions, whereas the sidechain of C-1748 is shorter and ending with a hydroxyl moiety, thus it was uncharged in the same environment (Fig. 7).

The main source of this seemingly unexpected behaviour of nitroacridines possibly lies within the structure of the ring systems of both compounds. The first reason for this apparent intercalation mutiny is the fact that these structures, i.e. C-283 and C-1748, are not flat. The 9-amino group, attached to the ring system, is in fact an imino group, whereas the proton—originally intended to reside at the 9-amino moiety—is located at the nitrogen atom embedded in the ring system, which was clearly evidenced by our previous NMR studies^6^ (Fig. 7). This results in two separate, aromatic benzene-type rings within the structure, whereas the whole system is slightly bent to the shape of a butterfly, which was displayed for the first time upon the crystallographic studies on Nitracrine^29^. Moreover, the oxygen atoms of the 1-nitro moiety are also not located within the plane of the attached aromatic ring. The –NO_2_ group is notably twisted, furtherly disturbing the already non-flat geometry of the ring system. As a result, the DNA/ligand stacking energies, expected to contribute to the stability of a resulting complex, in the case of nitroacridines could be simply too low to force an opening within the DNA. Even if the opening is being created, the resulting DNA:nitroacridine intercalation complex seems to be very unstable. Therefore, binding to the minor groove of a DNA duplex is being proposed—but not unambiguously evidenced—as the most reasonable mode of interaction that the dsDNA:nitroacridine system can afford, at least at pH ~ 6.0 and below. Under slightly basic conditions, the ring system of nitroacridines becomes negatively charged, which results in their notably lowered affinity to nucleic acids (Fig. 7).

As it was stated before, the unsymmetrical bisacridines (UAs) consist of two acridine-based ring systems (Fig. 1). Since one of them is identical to the ring system of C-1311 and considering the fact that C-1311 is a rather effective intercalating agent, one could expect that the UAs should be able to form at least ‘partial’ intercalation complexes, even taking into account that the second ring system is a copy of presumably non-intercalating (or possibly creating unstable intercalation complexes) Nitracrine or C-1748. The latter should be able to locate itself somewhere in a minor groove of the dsDNA, which would result in an interesting mode of UAs binding to DNA duplexes: half-intercalation (via C-1311 moiety), half minor-groove binding (via linker and nitroacridine moiety). Our NMR studies have proven that this concept is utterly wrong. Whereas, in theory, the UAs do indeed look like the perfect bis-intercalators, the reality is that they are not even half-intercalators, since it turned out that the presence of a C-1311’s aromatic moiety is not enough to force an intercalation mode for the binding of UAs into double-stranded DNA.

Taking into account the matters discussed above for both Symadex and the nitroacridines, including Nitracrine, it is finally much easier to explain why unsymmetrical bisacridines (UAs) do not act as dsDNA intercalators. The comparison of C-1305 and C-1311 has highlighted a huge role of an aminoalkyl sidechain during the dsDNA:ligand complex formation and its further stabilisation. In the case of UAs, regarding the potential intercalation, the linker and the nitroacridine moiety should be treated as a gigantic sidechain, attached to the imidazoacridinone ring system. This ‘sidechain’, albeit positively charged, may be either too big to specifically interact with the sugar-phosphate backbone of the DNA strands, or its structure might simply disfavour those interactions. Therefore, as a result, in this version of the story the sidechain serves as a destabilising agent after a potential intercalation event. Alternatively, the sidechain’s interactions within the minor groove of the DNA may effectively prevent the attached C-1311’s moiety from intercalation. Regardless of which one of the above statements is true—perhaps, in part, they are all true—the effect is the same: an intercalation event of UA molecule into dsDNA is not being observed in any case. Nevertheless, it should be noted that exchanging the nitroacridine moiety into a C-1305 ring system might possibly result in a creation of a potent UA bis-intercalator; more studies on this matter are on the way.

Double-stranded DNA is just one of the several forms that deoxyribonucleic acids are able assume. In the end, the lack of dsDNA/UAs interactions, resulting in the lack of UAs’ impact on dsDNA structure and function in a cell environment, may be considered as an advantage in some pharmacological scenarios. On the other hand, our preliminary NMR studies have strongly suggested that both C-1311 and UAs exhibit well-defined interactions with several DNA G-quadruplexes, which are currently regarded as very attractive molecular targets in anticancer therapy. These findings are perfectly in line with our previous reports, which displayed that UAs inhibit the expression of K-Ras in Panc-1 cells^1^, as well as c-Myc in H460 cells^30^. Advanced NMR studies on the resulting G4/UA complexes and the implications of their formation will be discussed in our future work.

## Methods

### Chemicals

5-Diethylaminoethylamino-8-hydroxyimidazoacridinone (C-1311, Symadex), 5-[[3-(dimethylamino)propyl]amino]-8-hydroxy-6H-v-triazolo[4,5,1-de]acridin-6-one (C-1305), 9-(2′-hydroxyethylamino)-4-methyl-1-nitroacridine, (C-1748), 1-nitro-9-[3′-(dimethylamino)propylamino]acridine (C-283, Ledakrin/Nitracrine), 9-{N-[(imidazo[4,5,1-de]-acridin-6-on-5-yl)aminopropyl]-N-methylaminopropylamino}-1′-nitroacridine × 1.5HCl (C-2028), 1-[3-(imidazo[4,5,1-de]-acridin-6-on-5-yl)aminopropyl]-4-[3′-(1′-nitroacridin-1-yl)-aminopropyl]piperazine × 4HCl (C-2041), 9-{N-[(8-hydroxyimidazo[4,5,1-de]-acridin-6-on-5-yl)aminopropyl]-N-methylaminopropylamino}-4′-methyl-1′-nitroacridine × 3HCl (C-2045), 9-{N-[(imidazo[4,5,1-de]-acridin-6-on-5-yl)aminopropyl]-N-methylaminopropylamino}-4′-methyl-1′-nitroacridine × 3HCl (C-2053) were synthesized at the Department of Pharmaceutical Technology and Biochemistry, Faculty of Chemistry, Gdansk University of Technology. All DNA sequences were purchased from Metabion, GmbH and additionally purified using Amicon Ultra 2 mL centrifugal filters provided by Merck. This process served to remove an impurity giving rise to very strong signals in the proton NMR spectra at around 1.28, 1.99, 3.21 and 8.60 ppm (triethylamine-acetate, used by the oligo supplier during HPLC purification).

### NMR sample preparation

Each oligo used in this study was fully self-complementary and thus the preparation of the duplex samples consisted simply of dissolving the purified material in an appropriate buffer. The optimal experimental conditions, lowering monoacridines’ and unsymmetrical bisacridines’ (UAs) tendency to self-association, while maintaining helical forms of short dsDNA oligomers were selected as 2.5 mM cacodylate buffer of pH 5.0, containing 10 mM NaCl. To prepare the samples of the dsDNA:ligand intercalation complexes, the ligand (monoacridine/UA, see Fig. 1) was added to the pre-mixed NMR sample from a concentrated stock solution in water, to reach to duplex:ligand molar ratio of 0.5, 1.0, 1.25 or 2.0, depending on the sample. The titration experiments were conducted by stepwise addition of the concentrated stock solutions of C-283 or C-1748 to a DNA sample; a single ligand portion corresponded to the 0.1 molar equivalent of the DNA hosting duplex.

### NMR spectra

All NMR spectra were collected using a 700 MHz Bruker Avance III HD spectrometer, equipped with a QCI CryoProbe. After three 8 bp DNA duplexes forming a single, well-defined complexes with C-1305 or C-1311 were identified (**D2**, **D3**, **B2**—see “Results”), a set of 2D spectra was recorded for resonance assignment for each of the free duplexes. It comprised the NOESY (150 ms mixing time) and TOCSY (60 ms spin-lock time) spectra measured at 5 °C in 90% H_2_O/10% D_2_O, as well as, the NOESY (150 and 400 ms mixing time), HC-HSQC, HP-COSY and DQF-COSY spectra acquired in 100% D_2_O at 5 °C. The resonance assignment process itself was performed using standard approaches^31^. For the assignment of the dsDNA/ligand complexes and for the identification of the DNA-ligand cross-peaks, the NOESY (150 ms mixing time), TOCSY (60 ms spin-lock time) and HC-HSQC spectra were recorded for the complexes at 5 °C, in both 90% H_2_O/10% D_2_O and 100% D_2_O.

### Molecular modelling

Molecular dynamics (MD) simulations were performed for the d(CGATATCG)_2_:C-1311, d(CCCTAGGG)_2_:C-1311 and d(CGCTAGCG)_2_:C-1305 intercalation complexes explicitly solvated in cubic boxes, with ~ 5500 TIP3P water molecules at 0.01 M concentration of NaCl. The force field parameters for the DNA octamers were taken from the latest iteration of CHARMM36 nucleic acid force field^32^. The parameters for C-1305 and C-1311 were taken from the latest version of CHARMM36 Generalized Force Field (CGenFF)^33^; the partial atomic charges of the ligand were calculated ab initio using GAUSSIAN09 software^34^ on the MP2/6-31G* level of theory. All energy minimizations and MD simulations were carried out using GROMACS 2020.4^35^. All the MD simulations were conducted using the leapfrog scheme with a time step of 2 fs. The particle mesh Ewald technique with a cutoff of 1 nm and grid spacing of approx. 0.1 nm was used to evaluate electrostatic forces^36^. The van der Waals interactions were calculated using Lennard–Jones potential with a cutoff of 1 nm. The simulations were conducted at a constant temperature of 278 K and at a constant pressure of 1 bar, using the weak coupling method^37^.

After obtaining the initial B-form of DNA duplexes from X3DNA 2.3^38^, one C-1305 or C-1311 molecule with a protonated tertiary nitrogen at the end of the sidechain was placed in a moderate proximity of a respective DNA duplex using VMD software^39^. After the appropriate energy minimization and 100 ns of MD-based initial equilibration with position restraints set on DNA and ligand molecule, each system was simulated for 1 ns. During this run, distance restraints corresponding to the NOE contacts between the aromatic protons of the ligand and the protons of the DNA were applied (Tables 2 and 4). This was done using the GROMACS implementation of the restraining potential which adds a quadratic penalty to the potential when a distance exceeds a lower or upper threshold (see Table S4 in the Supplementary Data). The same force constants of 1000 kJ mol^−1^ nm^−2^ were used for all restrained distances corresponding to DNA/ligand intermolecular contacts. The above described simulations resulted in a DR-driven intercalation of ligand molecules into the 5′-TA-3′/5′-TA-3′ site from the minor groove of DNA duplexes. Afterwards, the final frame from the each resulting trajectory was extracted. These frames were set as starting points for a 1 µs-long MD simulations described below, which were preceded by 100 ns of further equilibration with position restraints set on DNA and ligand molecules.

During this run, the systems were subjects to 19 (**D2L** and **D3L**) and 23 (**B2L**) distance restraints derived from the NOESY experiment of τ_m_ = 150 ms and another 14 (**D2L**) and 16 (**D3L**, **B2L**) distance restraints, strengthening the hydrogen bonds in Watson–Crick base pairs. All the base pairs were stabilized except the terminal G≡C pairs, since the G8 imino proton resonance was not observed in the ^1^H NMR spectra of either of the studied complexes. The distance restraining force constant was also equal to 1000 kJ mol^−1^ nm^−2^.

Cluster analysis was performed using the Daura method^40^ with a RMSD cutoff set to 0.2 nm.

### UV–VIS spectra

0.01 mM solutions of C-1311 and C-1305 were prepared and the DNA solutions were added in portions so as to obtain following rations: 0.25, 0.5, 1, 1.5, 2, 3, 5, 7 DNA in respect to C-1311 and C-1305. Concentration of ligand was kept constants. After each addition, UV–VIS spectra were collected in a quartz cuvette with an optical path length of 1 cm.

### Chemometric analysis

All sets of spectra were organised into matrices and centred. Afterwards, data matrices have underwent numerical decomposition into eigenvectors, using double principal component analysis (PCA) algorithm. Molar fractions of particular forms, calculated basing on selected eigenvectors, were used to prepare Scatchard plots. Dissociation constants were determined on the basis of Scatchard plot by piecewise regression.

The Scatchard plot was expressed as a function of ν/L versus v. As long as v was equal to n (number of identical and independent binding sites); n, L and M were calculated from previously determined mole fractions according to the Eq. (1):

1 $$v=(c-L)/M$$

where: v—average occupancy of DNA host by ligand molecule; M—total concentration of DNA; c—total ligand concentration; L—concentration of free ligand.

## Supplementary Information

Supplementary Information.

## Data Availability

Most of the data generated or analyzed during this study are included in this published article (and its Supplementary Data files). The full 2D NMR spectra and MD trajectories generated and analyzed during the current study are available from the corresponding author on reasonable request.
