# Supplementary material for: The interactions of monomeric acridines and unsymmetrical bisacridines (UAs) with DNA duplexes: an insight provided by NMR and MD studies
Source: Sci Rep. 2023 Mar 1;13:3431. doi: 10.1038/s41598-023-30587-y (PMC9977845; doi:10.1038/s41598-023-30587-y)
Supplement: Supplementary file 1 — Supplementary Information. [file 41598_2023_30587_MOESM1_ESM.pdf]

The interactions of monomeric acridines and unsymmetrical bisacridines (UAs) with DNA duplexes – an insight provided by NMR and MD studies

Tomasz Laskowski[1,*], Michał Kosno[1], Witold Andrałojć[2], Joanna E. Frackowiak[1], Julia Borzyszkowska-Bukowska[1], Paweł Szczeblewski[1], Nikola Radoń[1], Maria Świerżewska[1], Anna Woźny[3], Ewa Paluszkiewicz[1], Zofia Mazerska[1]

[1] Department of Pharmaceutical Technology and Biochemistry and BioTechMed Centre, Faculty of Chemistry, Gdańsk University of Technology, Gdańsk, Gabriela Narutowicza Str. 11/12, 80-233, Poland
[2] Institute of Bioorganic Chemistry, Polish Academy of Sciences, Poznań, Zygmunta Noskowskiego Str. 12/14, 61-704, Poland
[3] Faculty of Applied Physics and Mathematics, Gdańsk University of Technology, Gdańsk, Gabriela Narutowicza Str. 11/12, 80-233, Poland

* tomasz.laskowski@pg.edu.pl

SUPPLEMENTARY DATA

CONTENTS:

Figure S1. *The H6/H8-H1' NOESY-walks for the A and B strands of dsDNA duplex, traced for the d(CGATATCG)$_2$:C-1311 complex (**D2L**).* 3
Figure S2. *The H6/H8-H1' NOESY-**walks** for the A and B strands of dsDNA duplex, traced for the d(CCCTAGGG)$_2$:C-1311 complex (D3L).* 4
Figure S3. *The H6/H8-H1' NOESY-walks for the A and B strands of dsDNA duplex, traced for the d(CGCTAGCG)$_2$:C-1305 complex (**B2L**).* 5
Figure S4. *Codenames assigned to the protons of C-1311 (Symadex) and C-1305.* 6
***Table S1**. ^{1}H NMR chemical shifts (δ [ppm], referenced to TSP residual signal set to 0.000 ppm) of the protons of the ligands, as well as the protons of the DNA duplexes forming the d(CGATATCG)$_2$:C-1311 (D2L), d(CCCTAGGG)$_2$:C-1311 (**D3L**) and d(CGCTAGCG)$_2$:C-1305 (**B2L**) complexes.* 6
***Table S2**. ^{1}H NMR chemical shifts (δ [ppm], referenced to TSP residual signal set to 0.000 ppm) of the protons of the free d(CGCTAGCG)$_2$ DNA duplex.* 8
Figure S5. *UV-VIS and chemometric analysis of C-1305 and C-1311 binding to DNA duplexes: spectra resulting from titration.* 9
Figure S6. *A comparison of the UV-VIS spectra of the free monoacridines, i.e. C-1305 and C-1311, and bound to a dsDNA sequence with 1:1 mol/mol stoichiometry.* 9
Figure S7. *UV-VIS and chemometric analysis of C-1305 and C-1311 binding to DNA duplexes: spectra of the principal components.* 10
Figure S8. *UV-VIS and chemometric analysis of C-1305 and C-1311 binding to DNA duplexes: molar fractions.* 10
Figure S9. *UV-VIS and chemometric analysis of C-1305 and C-1311 binding to DNA duplexes: Scatchard plots.* 11
Table S3. *Interactions of unsymmetrical bisacridines (UAs), i.e. C-2028, C-2041, C-2045, and C-2053, with dsDNA duplexes.* 11
Table S4. *The definition of distance restraints used in molecular dynamics simulations.* 11
Figure S10. *Titration experiments, performed for Nitracrine and C-1748, using d(CGATATCG)$_2$ (**D2**) dsDNA hosting duplex.* 12

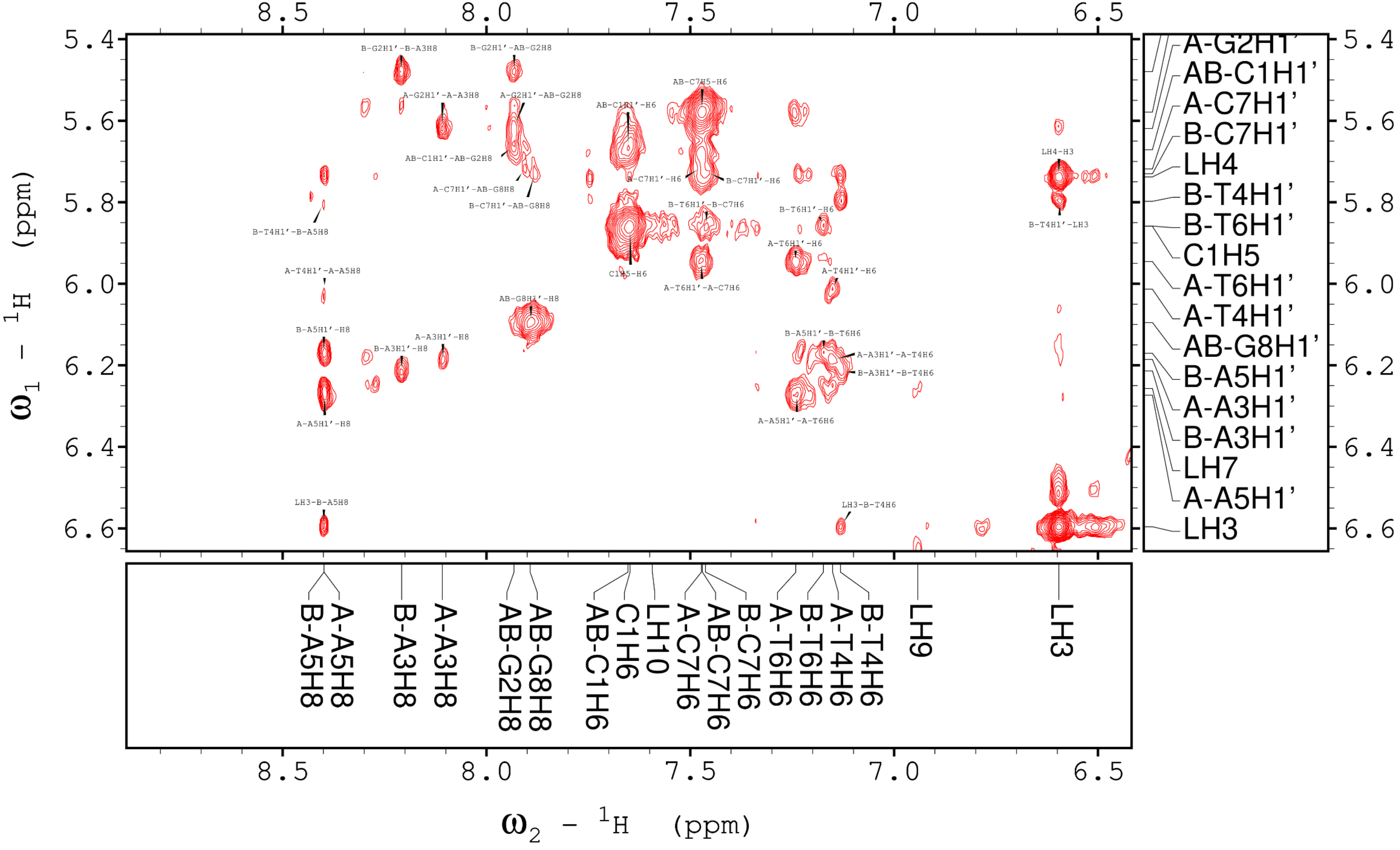

Figure S1. The H6/H8-H1' NOESY-walks for the A and B strands of dsDNA duplex, traced for the d(CGATATCG)$_2$:C-1311 complex (**D2L**).

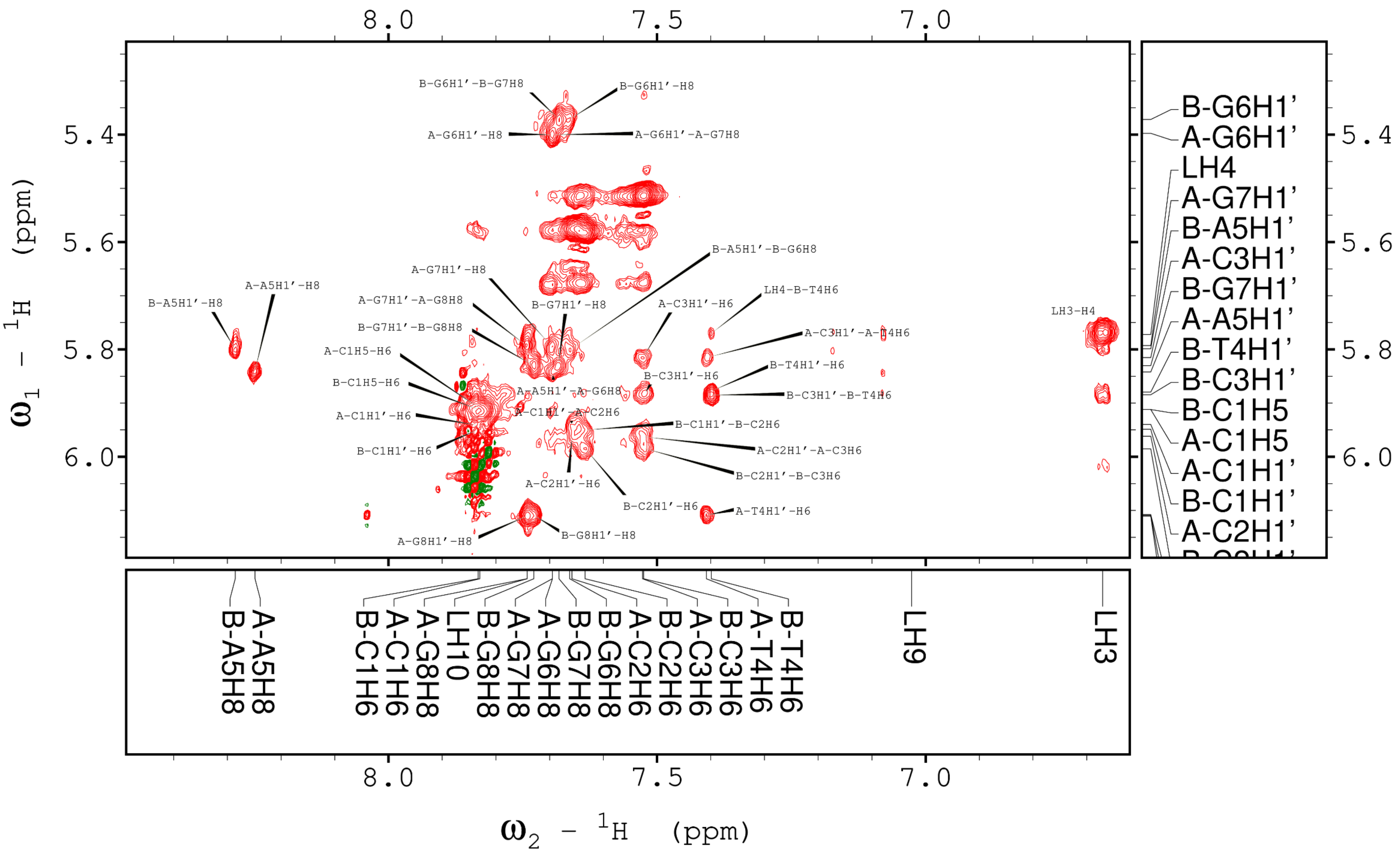

Figure S2. The H6/H8-H1' NOESY-walks for the A and B strands of dsDNA duplex, traced for the d(CCCTAGGG)$_2$:C-1311 complex (**D3L**).

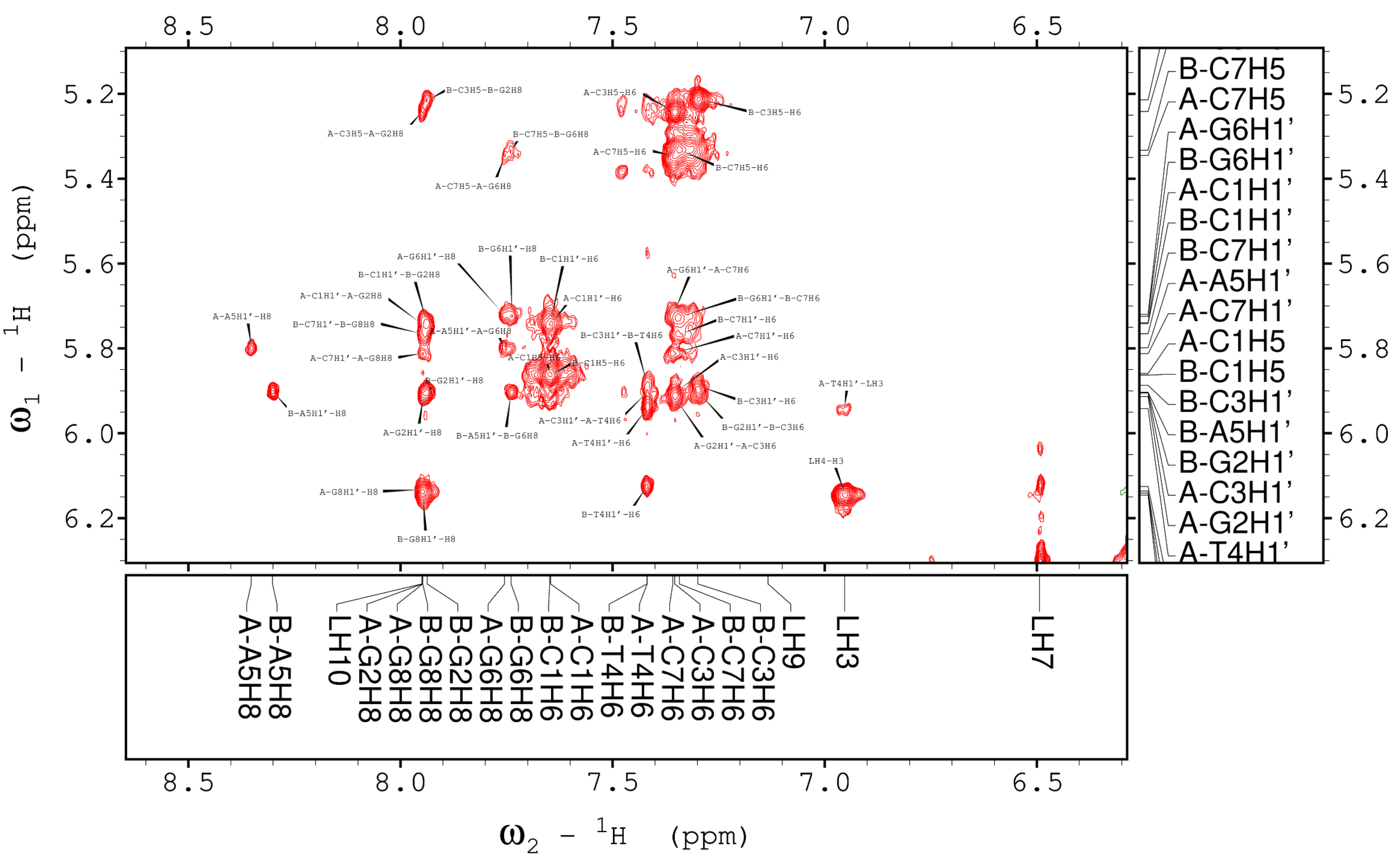

Figure S3. The H6/H8-H1’ NOESY-walks for the A and B strands of dsDNA duplex, traced for the d(CGCTAGCG)$_2$:C-1305 complex (**B2L**).

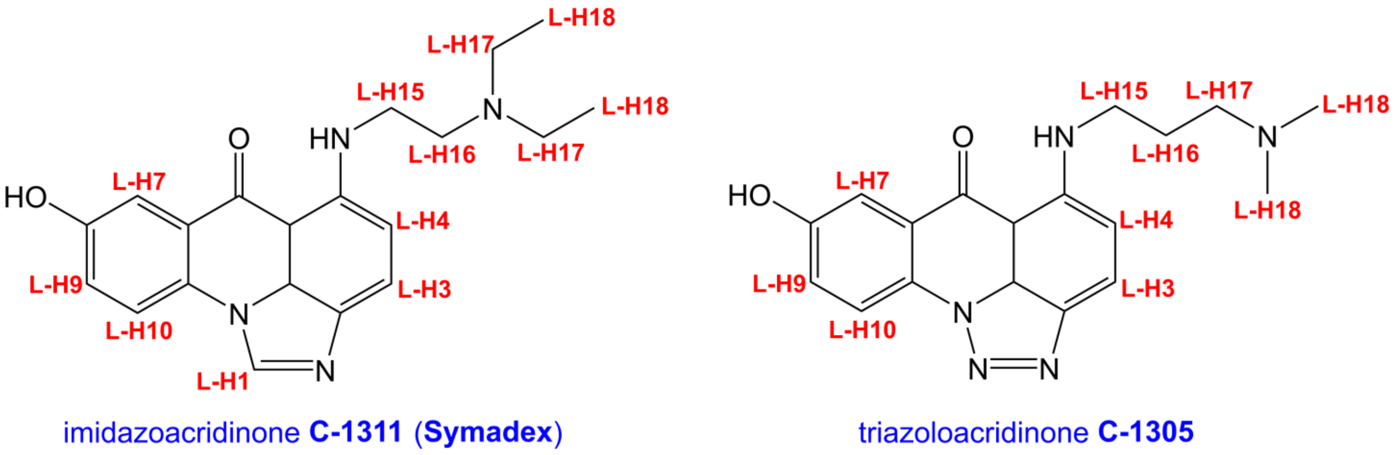

Figure S4. Codenames assigned to the protons of C-1311 (Symadex) and C-1305.

Table S1. ^{1}H NMR chemical shifts (δ [ppm], referenced to TSP residual signal set to 0.000 ppm) of the protons of the ligands, as well as the protons of the DNA duplexes forming the d(CGATATCG)$_2$:C-1311 (**D2L**), d(CCCTAGGG)$_2$:C-1311 (**D3L**) and d(CGCTAGCG)$_2$:C-1305 (**B2L**) complexes. Deoxyribose protons H4', H5' and H5'' were not listed due to severe superpositions of proton resonances. Chemical shifts of the protons of the free **D2** and **D3** duplexes were given in the Reference 11 (see the main manuscript); chemical shifts of the protons of the free **B2** duplex were listed in Table S2.

d(CGATATCG)$_2$:C-1311 complex (D2L)		d(CCCTAGGG)$_2$:C-1311 complex (D3L)		d(CGCTAGCG)$_2$:C-1305 complex (B2L)	
proton	δ (ppm)	proton	δ (ppm)	proton	δ (ppm)
ligand (C-1311)		ligand (C-1311)		ligand (C-1305)	
L-H1	8.403	L-H1	8.497	L-H3	6.953
L-H3	6.596	L-H3	6.670	L-H4	6.146
L-H4	5.738	L-H4	5.772	L-H7	6.494
L-H7	6.257	L-H7	6.417	L-H9	7.134
L-H9	6.942	L-H9	7.026	L-H10	7.950
L-H10	7.593	L-H10	7.742	L-H15	3.418
L-H15	3.611	L-H15	3.576	L-H16	3.273
L-H16	3.458	L-H16	3.450	L-H17	3.273
L-H17	3.365	L-H17	3.323	L-H18	2.947
L-H18	1.426	L-H18	1.360		
DNA duplex					
A-C1H1'	5.671	A-C1H1'	5.940	A-C1H1'	5.740
A-C1H2''	2.390	A-C1H2'	2.214	A-C1H2'	2.012
A-C1H2'	2.378	A-C1H2''	2.242	A-C1H2''	2.434
A-C1H3'	4.684	A-C1H3'	4.653	A-C1H3'	4.716
A-C1H5	5.859	A-C1H5	5.912	A-C1H5	5.859
A-C1H6	7.653	A-C1H6	7.830	A-C1H6	7.646
B-C1H1'	5.671	B-C1H1'	5.949	B-C1H1'	5.741
B-C1H2''	2.390	B-C1H2'	2.210	B-C1H2'	2.004
B-C1H2'	2.378	B-C1H2''	2.235	B-C1H2''	2.424
B-C1H3'	4.684	B-C1H3'	4.658	B-C1H3'	4.716
B-C1H5	5.859	B-C1H5	5.912	B-C1H5	5.863
B-C1H6	7.653	B-C1H6	7.833	B-C1H6	7.648
A-G2H1'	5.619	A-C2H1'	5.962	A-G2H1'	5.914
A-G2H2''	2.742	A-C2H2'	2.313	A-G2H2'	2.664
A-G2H2'	2.722	A-C2H2''	2.466	A-G2H2''	2.705
A-G2H3'	4.990	A-C2H3'	4.834	A-G2H3'	4.979
A-G2H8	7.933	A-C2H6	7.659	A-G2H8	7.948

B-G2H1'	5.480	B-C2H1'	5.985	B-G2H1'	5.904
B-G2H2"	2.742	B-C2H2'	2.232	B-G2H2'	2.666
B-G2H2'	2.722	B-C2H2"	2.472	B-G2H2"	2.704
B-G2H3'	4.990	B-C2H3'	4.853	B-G2H3'	4.974
B-G2H8	7.933	B-C2H6	7.634	B-G2H8	7.937
A-A3H1'	6.186	A-C3H1'	5.815	A-C3H1'	5.905
A-A3H2"	2.833	A-C3H2'	2.040	A-C3H2'	2.459
A-A3H2'	2.537	A-C3H2"	2.433	A-C3H2"	2.702
A-A3H3'	4.948	A-C3H3'	4.701	A-C3H3'	4.765
A-A3H8	8.108	A-C3H6	7.527	A-C3H5	5.242
B-A3H1'	6.214	B-C3H1'	5.884	A-C3H6	7.354
B-A3H2"	2.902	B-C3H2'	2.019	B-C3H1'	5.887
B-A3H2'	2.586	B-C3H2"	2.450	B-C3H2'	2.428
B-A3H3'	5.039	B-C3H3'	4.794	B-C3H2"	2.691
B-A3H8	8.208	B-C3H6	7.525	B-C3H3'	4.658
A-T4CH3	1.190	A-T4CH3	1.489	B-C3H5	5.214
A-T4H1'	6.013	A-T4H1'	6.108	B-C3H6	7.299
A-T4H2"	2.538	A-T4H2'	2.592	A-T4CH3	1.641
A-T4H2'	2.491	A-T4H2"	2.606	A-T4H1'	5.942
A-T4H3'	4.972	A-T4H3'	4.998	A-T4H2'	2.487
A-T4H6	7.151	A-T4H6	7.408	A-T4H2"	2.617
B-T4CH3	1.388	B-T4CH3	1.644	A-T4H3'	5.018
B-T4H1'	5.798	B-T4H1'	5.880	A-T4H6	7.418
B-T4H2"	2.386	B-T4H2'	2.473	B-T4CH3	1.465
B-T4H2'	2.328	B-T4H2"	2.488	B-T4H1'	6.125
B-T4H3'	4.990	B-T4H3'	5.007	B-T4H2'	2.477
B-T4H6	7.132	B-T4H6	7.399	B-T4H2"	2.615
A-A5H1'	6.273	A-A5H1'	5.842	B-T4H3'	5.018
A-A5H2"	2.952	A-A5H2'	2.770	B-T4H6	7.419
A-A5H2'	2.815	A-A5H2"	2.806	A-A5H1'	5.798
A-A5H3'	4.993	A-A5H3'	4.979	A-A5H2'	2.783
A-A5H8	8.398	A-A5H8	8.249	A-A5H2"	2.802
B-A5H1'	6.171	B-A5H1'	5.799	A-A5H3'	4.963
B-A5H2"	2.935	B-A5H2'	2.712	A-A5H8	8.352
B-A5H2'	2.699	B-A5H2"	2.793	B-A5H1'	5.904
B-A5H3'	4.938	B-A5H3'	4.917	B-A5H2'	2.809
B-A5H8	8.399	B-A5H8	8.285	B-A5H2"	2.837
A-T6CH3	1.188	A-G6H1'	5.398	B-A5H3'	5.001
A-T6H1'	5.946	A-G6H2'	2.547	B-A5H8	8.302
A-T6H2"	2.415	A-G6H2"	2.579	A-G6H1'	5.720
A-T6H2'	2.080	A-G6H3'	4.954	A-G6H2'	2.556
A-T6H3'	4.859	A-G6H8	7.695	A-G6H2"	2.574
A-T6H6	7.241	B-G6H1'	5.372	A-G6H3'	4.976
B-T6CH3	1.246	B-G6H2'	2.547	A-G6H8	7.755
B-T6H1'	5.859	B-G6H2"	2.572	B-G6H1'	5.725
B-T6H2"	2.407	B-G6H3'	4.944	B-G6H2'	2.557
B-T6H2'	2.028	B-G6H8	7.663	B-G6H2"	2.574
B-T6H3'	4.817	A-G7H1'	5.793	B-G6H3'	4.975
B-T6H6	7.174	A-G7H2'	2.584	B-G6H8	7.739
A-C7H1'	5.719	A-G7H2"	2.743	A-C7H1'	5.812
A-C7H2"	2.400	A-G7H3'	4.981	A-C7H2'	2.371
A-C7H2'	2.322	A-G7H8	7.695	A-C7H2"	2.569
A-C7H3'	4.825	B-G7H1'	5.830	A-C7H3'	4.853
A-C7H5	5.579	B-G7H2'	2.579	A-C7H5	5.345
A-C7H6	7.470	B-G7H2"	2.763	A-C7H6	7.358
B-C7H1'	5.730	B-G7H3'	4.981	B-C7H1'	5.766
B-C7H2"	2.400	B-G7H8	7.683	B-C7H2'	2.359
B-C7H2'	2.322	A-G8H1'	6.109	B-C7H2"	2.569
B-C7H3'	4.825	A-G8H2'	2.495	B-C7H3'	4.835
B-C7H5	5.579	A-G8H2"	2.330	B-C7H5	5.333
B-C7H6	7.470	A-G8H3'	4.646	B-C7H6	7.342

**A-G8H1'**	6.095	**A-G8H8**	7.742	**A-G8H1'**	6.136
**A-G8H2"**	2.337	**B-G8H1'**	6.111	**A-G8H2'**	2.656
**A-G8H2'**	2.672	**B-G8H2'**	2.505	**A-G8H2"**	2.363
**A-G8H3'**	4.691	**B-G8H2"**	2.337	**A-G8H3'**	4.687
**A-G8H8**	7.893	**B-G8H3'**	4.646	**A-G8H8**	7.948
**B-G8H1'**	6.095	**B-G8H8**	7.730	**B-G8H1'**	6.141
**B-G8H2"**	2.337			**B-G8H2'**	2.645
**B-G8H2'**	2.672			**B-G8H2"**	2.357
**B-G8H3'**	4.691			**B-G8H3'**	4.687
**B-G8H8**	7.893			**B-G8H8**	7.948

Table S2. ^{1}H NMR chemical shifts (δ [ppm], referenced to TSP residual signal set to 0.000 ppm) of the protons of the free d(CGCTAGCG)$_2$ DNA duplex.

proton	δ (ppm)	proton	δ (ppm)
**C1H1'**	5.634	**A5H1'**	5.891
**C1H2'**	1.904	**A5H2**	7.188
**C1H2"**	2.324	**A5H2'**	2.610
**C1H3'**	4.581	**A5H2"**	2.751
**C1H4'**	3.934	**A5H3'**	4.910
**C1H5**	5.785	**A5H4'**	4.275
**C1H5'**	3.585	**A5H5'**	3.928
**C1H5"**	3.594	**A5H5"**	4.000
**C1H6**	7.536	**A5H8**	8.110
**G2H1'**	5.820	**G6H1'**	5.573
**G2H2'**	2.590	**G6H2'**	2.365
**G2H2"**	2.622	**G6H2"**	2.469
**G2H3'**	4.860	**G6H3'**	4.820
**G2H4'**	4.244	**G6H4'**	4.236
**G2H5'**	3.869	**G6H5'**	4.040
**G2H5"**	3.976	**G6H5"**	4.057
**G2H8**	7.874	**G6H8**	7.591
**C3H1'**	5.847	**C7H1'**	5.580
**C3H2'**	1.941	**C7H2'**	1.746
**C3H2"**	2.376	**C7H2"**	2.195
**C3H3'**	4.644	**C7H3'**	4.657
**C3H4'**	4.114	**C7H4'**	4.000
**C3H5**	5.237	**C7H5**	5.192
**C3H5'**	4.037	**C7H5'**	3.946
**C3H5"**	4.049	**C7H5"**	3.960
**C3H6**	7.331	**C7H6**	7.171
**T4H1'**	5.429	**G8H1'**	6.020
**T4H2'**	2.035	**G8H2'**	2.478
**T4H2"**	2.331	**G8H2"**	2.195
**T4H3'**	4.737	**G8H3'**	4.527
**T4H4'**	4.006	**G8H4'**	4.035
**T4H5'**	3.925	**G8H5'**	3.907
**T4H5"**	3.959	**G8H5"**	3.919
**T4H6**	7.308	**G8H8**	7.812

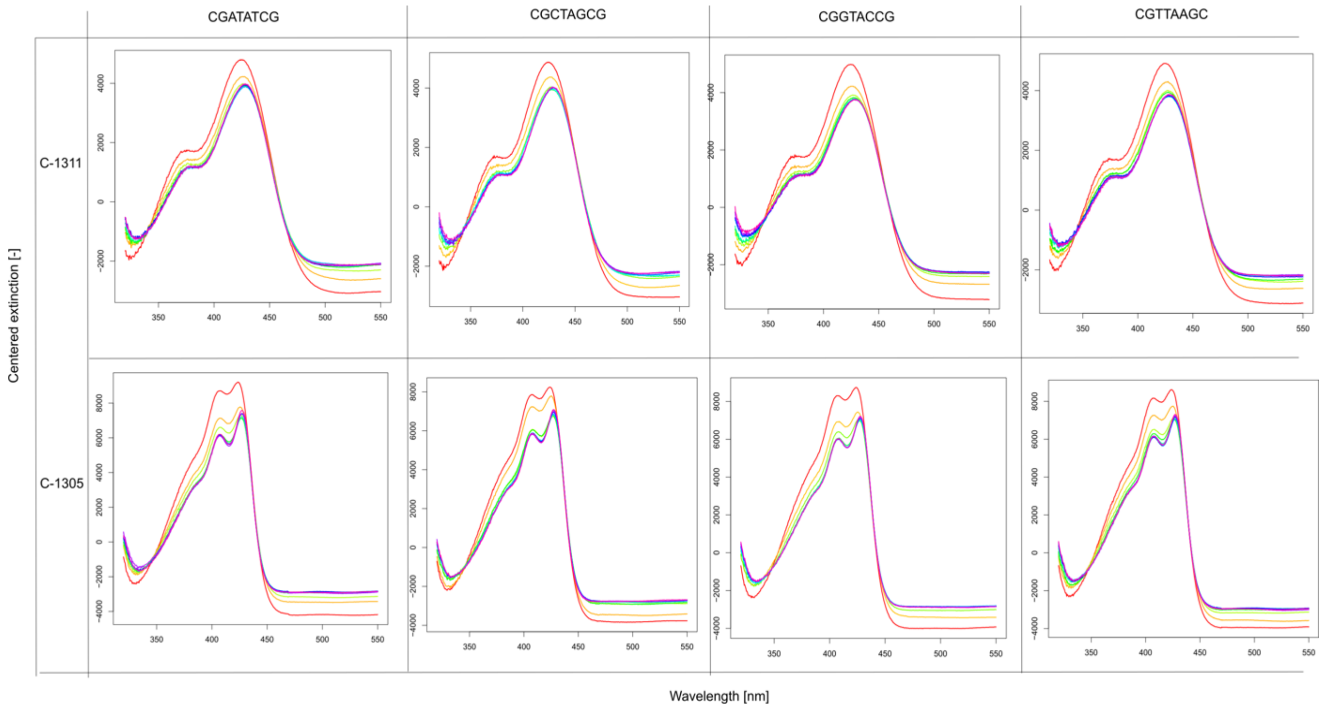

Figure S5. UV-VIS and chemometric analysis of C-1305 and C-1311 binding to DNA duplexes: spectra resulting from titration.

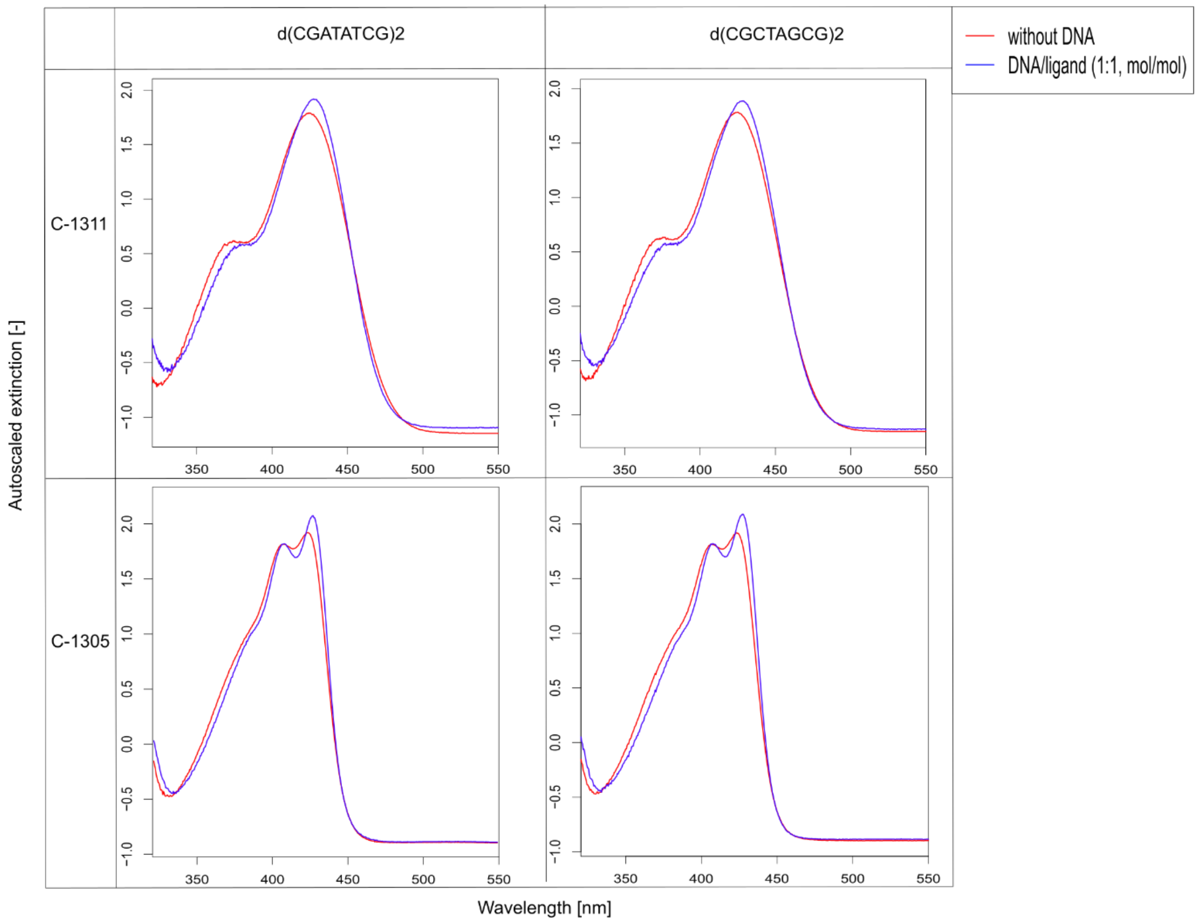

Figure S6. A comparison of the UV-VIS spectra of the free monoacridines, i.e. C-1305 and C-1311 (red line), and bound to a dsDNA sequence with 1:1 mol/mol stoichiometry (blue line).

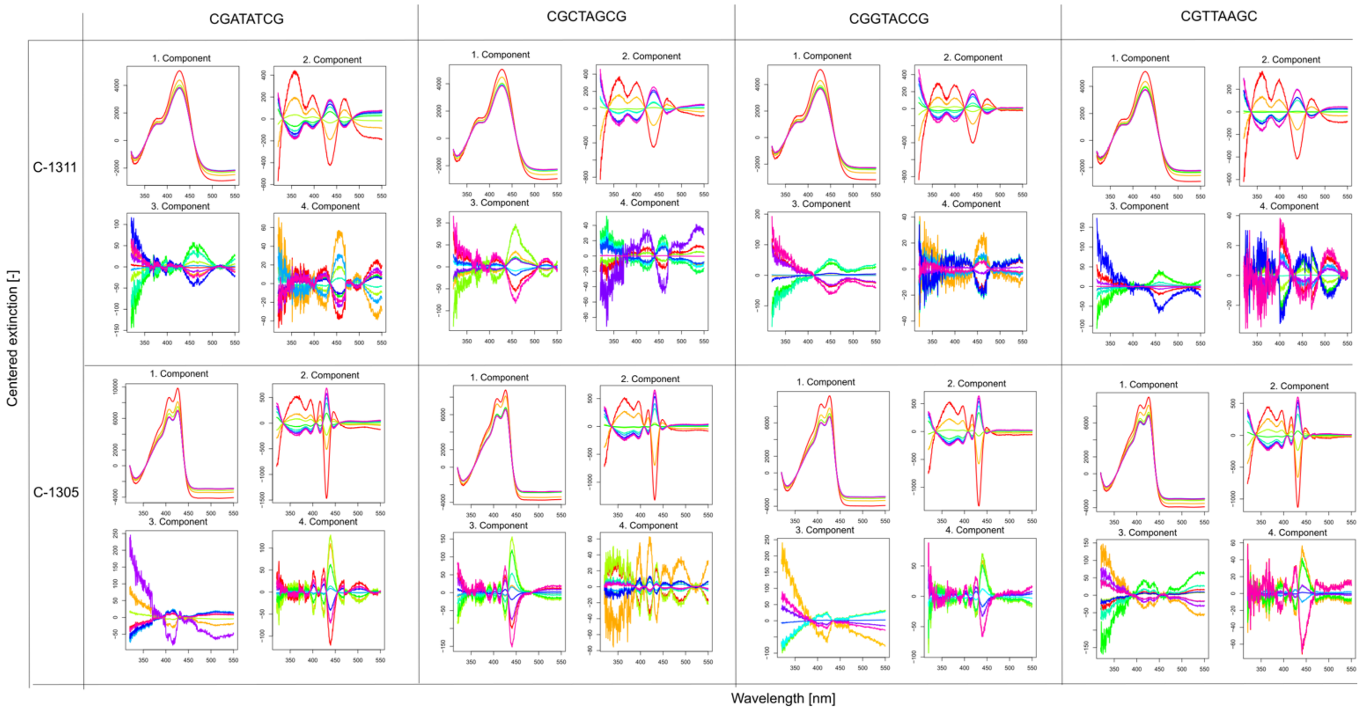

Figure S7. UV-VIS and chemometric analysis of C-1305 and C-1311 binding to DNA duplexes: spectra of the principal components.

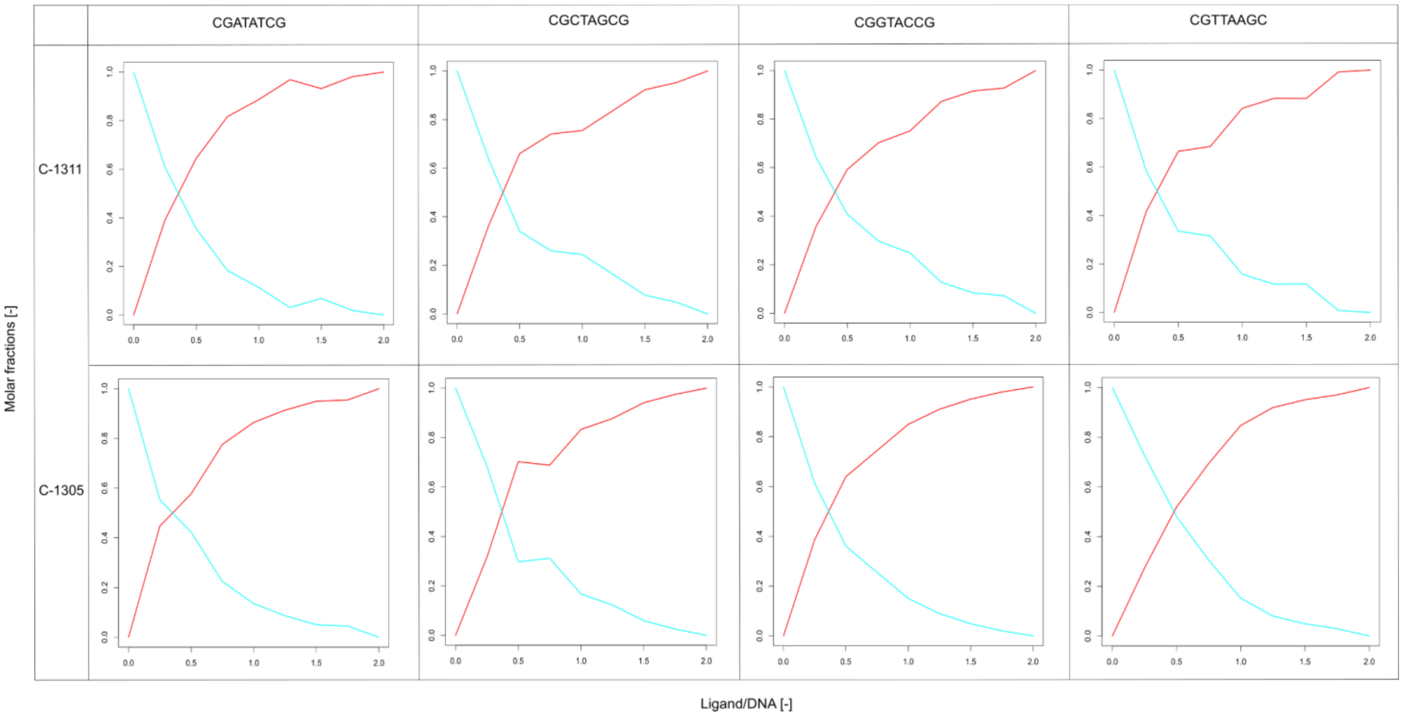

Figure S8. UV-VIS and chemometric analysis of C-1305 and C-1311 binding to DNA duplexes: molar fractions.

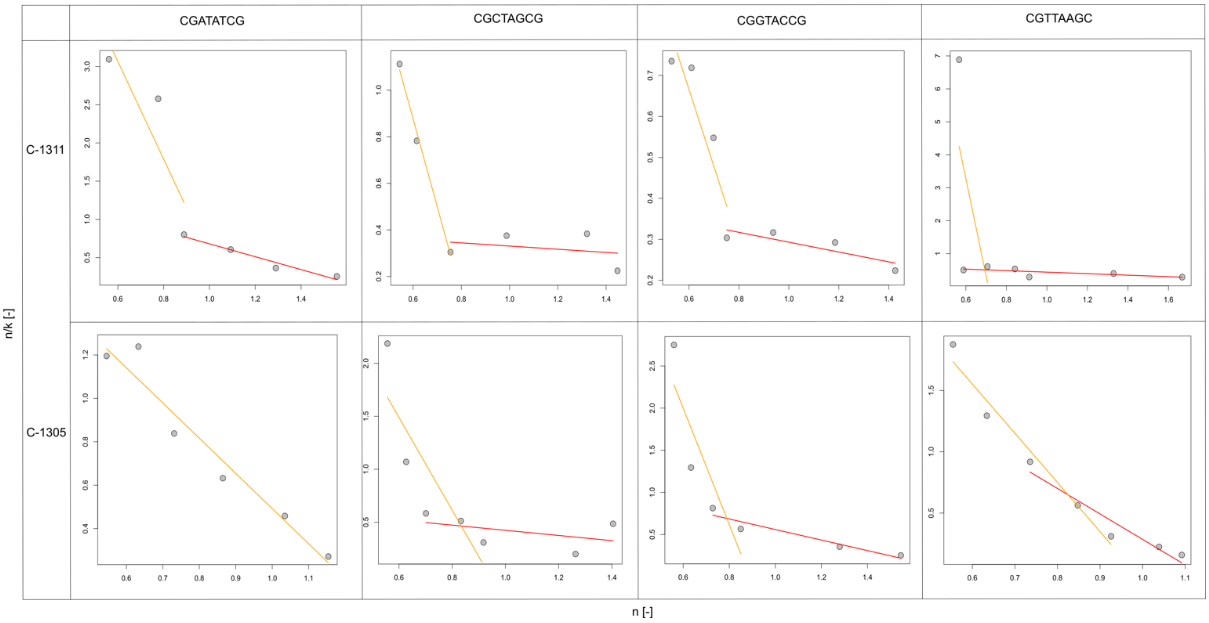

Figure S9. UV-VIS and chemometric analysis of C-1305 and C-1311 binding to DNA duplexes: Scatchard plots.

Table S3. Interactions of unsymmetrical bisacridines (UAs), i.e. C-2028, C-2041, C-2045, and C-2053, with dsDNA duplexes.

Oligonucleotide codename	Sequence 5'-3'	Binding site in the case of C-2028	Binding site in the case of C-2041	Binding site in the case of C-2045	Binding site in the case of C-2053
**U1**	CGATGCATCG	-	-	-	-
**U2**	CGTAGCTACG	-	-	-	-
**U3**	CTGACGTCAG	-	-	-	-
**U4**	CTAGCGCTAG	-	-	-	-

Table S4. The definition of distance restraints used in molecular dynamics simulations. When the distance between two protons fits between 'down' and 'up1' values: no potential is being used to bring the respective protons closer; when the distance fits between 'up1' and 'up2' or its value is lower than 'down': a quadratic potential is being added; when the distance exceeds 'up2' limit: the potential becomes linear.

Distance restraint definition:	down [nm]	up1 [nm]	up2 [nm]
**dsDNA/ligand intermolecular contact**			
**strong**	0.2000	0.3250	0.4250
**medium**	0.2500	0.4500	0.5500
**weak**	0.3000	0.6000	0.7000
**Watson-Crick hydrogen bonds**			
**A=T hydrogen bond: AN1-TH3**	0.1760	0.2160	0.3160
**A=T hydrogen bond: AH6-TO4**	0.1850	0.2250	0.3250
**G≡C hydrogen bond: GH2-CO2**	0.1680	0.2080	0.3080
**G≡C hydrogen bond:GH1-CN3**	0.1800	0.2200	0.3200
**G≡C hydrogen bond: GO6-CH4**	0.1800	0.2200	0.3200

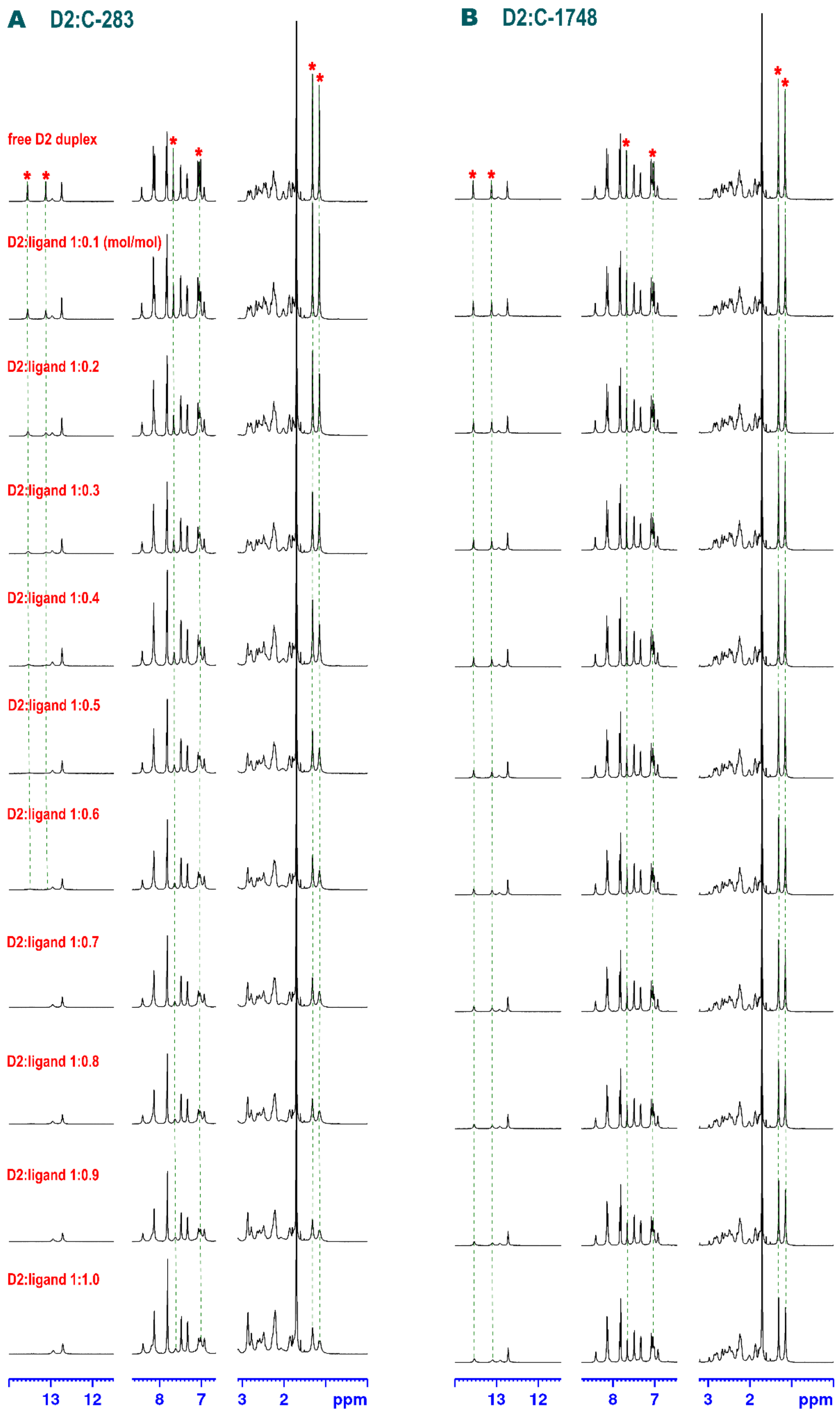

Figure S10. Titration experiments, performed for Nitracrine (panel **A**) and C-1748 (panel **B**), using d(CGATATCG)$_2$ (**D2**) dsDNA hosting duplex. Protons of the DNA, which were most notably affected by a ligand's presence, i.e. imino protons T4 & T6 and unexchangeable protons A3H2, A5H2, T4H6, T6H6, T4CH$_3$ and T6CH$_3$ were marked with red asterisks (*).
